# Efficient toolkit implementing best practices for principal component analysis of population genetic data

**DOI:** 10.1093/bioinformatics/btaa520

**Published:** 2020-05-16

**Authors:** Florian Privé, Keurcien Luu, Michael G B Blum, John J McGrath, Bjarni J Vilhjálmsson

**Affiliations:** National Centre for Register-Based Research, Aarhus University, Aarhus 8210, Denmark; Laboratoire TIMC-IMAG, UMR 5525, Univ. Grenoble Alpes, La Tronche 38700, France; Laboratoire TIMC-IMAG, UMR 5525, Univ. Grenoble Alpes, La Tronche 38700, France; Laboratoire TIMC-IMAG, UMR 5525, Univ. Grenoble Alpes, La Tronche 38700, France; OWKIN France, Paris 75010, France; National Centre for Register-Based Research, Aarhus University, Aarhus 8210, Denmark; Queensland Brain Institute, University of Queensland, St. Lucia, 4072 Queensland, Australia; Queensland Centre for Mental Health Research, The Park Centre for Mental Health, Wacol, 4076 Queensland, Australia; National Centre for Register-Based Research, Aarhus University, Aarhus 8210, Denmark

## Abstract

**Motivation:**

Principal component analysis (PCA) of genetic data is routinely used to infer ancestry and control for population structure in various genetic analyses. However, conducting PCA analyses can be complicated and has several potential pitfalls. These pitfalls include (i) capturing linkage disequilibrium (LD) structure instead of population structure, (ii) projected PCs that suffer from shrinkage bias, (iii) detecting sample outliers and (iv) uneven population sizes. In this work, we explore these potential issues when using PCA, and present efficient solutions to these. Following applications to the UK Biobank and the 1000 Genomes project datasets, we make recommendations for best practices and provide efficient and user-friendly implementations of the proposed solutions in R packages bigsnpr and bigutilsr.

**Results:**

For example, we find that PC19–PC40 in the UK Biobank capture complex LD structure rather than population structure. Using our automatic algorithm for removing long-range LD regions, we recover 16 PCs that capture population structure only. Therefore, we recommend using only 16–18 PCs from the UK Biobank to account for population structure confounding. We also show how to use PCA to restrict analyses to individuals of homogeneous ancestry. Finally, when projecting individual genotypes onto the PCA computed from the 1000 Genomes project data, we find a shrinkage bias that becomes large for PC5 and beyond. We then demonstrate how to obtain unbiased projections efficiently using bigsnpr. Overall, we believe this work would be of interest for anyone using PCA in their analyses of genetic data, as well as for other omics data.

**Availability and implementation:**

R packages bigsnpr and bigutilsr can be installed from either CRAN or GitHub (see https://github.com/privefl/bigsnpr). A tutorial on the steps to perform PCA on 1000G data is available at https://privefl.github.io/bigsnpr/articles/bedpca.html. All code used for this paper is available at https://github.com/privefl/paper4-bedpca/tree/master/code.

**Supplementary information:**

Supplementary data are available at *Bioinformatics* online.

## 1 Introduction

Principal component analysis (PCA) has been widely used in genetics for many years and in many contexts. For instance, adding PCs as covariates is routinely used to adjust for population structure in Genome-Wide Association Studies (GWAS) (Novembre and Stephens, 2008; Price *et al.*, 2006). PCA has also been used to detect loci under selection (Galinsky *et al.*, 2016; Luu *et al.*, 2017; Privé *et al.*, 2020) and in heritability analyses (Loh *et al.*, 2015a; Yang *et al.*, 2010). Recently, the advent of large population-scale genetic datasets, such as the UK Biobank data, has prompted research on developing scalable algorithms to compute PCA on very large data (Bycroft *et al.*, 2018). It is now possible to efficiently approximate PCA on very large datasets thanks to software such as FastPCA (fast mode of EIGENSOFT), FlashPCA2, PLINK 2.0 (approx mode), bigstatsr/bigsnpr, TeraPCA and ProPCA (Abraham *et al.*, 2017; Agrawal *et al.*, 2019; Bose *et al.*, 2019; Chang *et al.*, 2015; Galinsky *et al.*, 2016; Privé *et al.*, 2018).

However, in practice, conducting PCA on genotype data to capture population structure consists of more steps than simply performing singular value decomposition (SVD) on the genotype matrix. These steps include removing related individuals, pruning variants in linkage disequilibrium (LD), and excluding outlier samples that can suggest poor genotyping quality or distant relatedness. Some genetic analyses may also require to restrict to individuals of homogeneous ancestry. Many pitfalls related to PCA of genotype data have been documented and none of the currently available software address all of these. In the following, we outline these pitfalls and explain when they are relevant. First, some of the PCs may capture LD structure rather than population structure (Abdellaoui *et al.*, 2013; Price *et al.*, 2008; Privé *et al.*, 2018; Zou *et al.*, 2010). Including PCs that capture LD as covariates in genetic analyses can lead to reduced power for detecting genetic associations within these LD regions (Zou *et al.*, 2010). Second, another issue may arise when projecting a new study dataset to the PCA space computed from a reference dataset: projected PCs are shrunk toward 0 in the new dataset (Lee *et al.*, 2010; Wang *et al.*, 2015; Zhang *et al.*, 2020). This shrinkage makes it potentially dangerous to use the projected PCs for analyses, such as PC regression, ancestry detection and correction for ancestry. This same issue also arises when projecting individuals from the same dataset that were discarded from the PCA computation (e.g. related individuals). Third, PC scores may capture outliers that are due to family structure, population structure or other reasons; it might be beneficial to detect and remove these individuals to maximize the population structure captured by PCA (in the case of removing a few outliers) or to restrict analyses to genetically homogeneous samples (e.g. ‘White British’ people in the UK Biobank). Finally, efficient methods for PCA use approximations, which can results in some lack of precision of computed PCs. This potential issue has been demonstrated for software such as FastPCA and PLINK 2.0, but not for FlashPCA2 and bigstatsr/bigsnpr (Abraham *et al.*, 2017; Privé *et al.*, 2018). An overview of existing methods with their respective advantages and limitations is presented in Table 1.

**Table 1.** Overview of existing methods

Analysis	Method and/or software	Citation	Advantages	Current limitations
PCA	bigstatsr/bigsnpr	Privé *et al.* (2018)	Fast and accurate+handle dosages+thinning options directly included	Own format without missing values (fast functions are available for converting and imputing)
	FlashPCA2	Abraham *et al.* (2017)	Fast and accurate	Not parallelized
	PLINK 2.0 (reimplementation of FastPCA)	Galinsky *et al.* (2016) and Chang *et al.* (2015)	Fast	Possible lack of accuracy (Abraham *et al.*, 2017; Privé *et al.*, 2018)
Detection of outlier samples	‘6 SDs from the mean’ in EIGENSOFT	Patterson *et al.* (2006)	Simple	Assumes a Gaussian distribution
Detection of homogeneous samples	R package aberrant	Bellenguez *et al.* (2012)	Robust	Uses only two statistics at once
Projection of new individuals onto reference PCA space	Simple projection (multiplication by loadings)	Dey and Lee (2019)	Simple	Shrinkage biased
	Bias-adjusted projection in R package hdpca		Independent of new samples	Assumes same shrinkage for all individuals+model-based+need all eigenvalues of reference
	Augmentation, Decomposition and Procrustes (ADP) transformation in LASER 2.0	Wang *et al.* (2015)	Accurate	Slow (a new PCA for each new sample)
	Online ADP (OADP) in python package FRAPOSA	Zhang *et al.* (2020)	Much faster than ADP	Does not work for related individuals (Section 4.3)

## 2 Approach

For this article, we derive implementations of truncated PCA and other useful functions for e.g. performing LD thinning and computing various statistics. We make these available in a new release of R package bigsnpr (v1.0.0); what differs from previously available functions presented in Privé *et al.* (2018) is that these new functions can be used directly on PLINK bed/bim/fam files with some missing values. We use these new functions to analyze the UK Biobank data, and show that these functions are both very fast and easy to use. We also point out that many PCs currently reported by the UK Biobank capture LD structure instead of population structure. Interestingly, subsetting the UK Biobank data enables to get more PCs that capture population structure than when using the whole sample (∼40 instead of ∼16). Then, we project the other individuals that were not used in the PCA calculation, show that this projection is biased and provide an efficient solution to get unbiased projections instead. Finally, we explore options to detect outlier samples in PCA, either a few outlier samples that may correspond to e.g. batch effects or distant family structure, or when the goal is to restrict the data to individuals of homogeneous ancestry.

## 3 Materials and methods

### 3.1 Efficient implementation of PCA for genotype data

When there is no missing value, we compute the truncated SVD $U\Delta V^{T}$ of the scaled genotype matrix of diploid individuals ${\tilde{G}}_{i,j}=\frac{G_{i,j}-2\hat{f_{j}}}{\sqrt{2\hat{f_{j}}(1-\hat{f_{j}})}}$, where $G_{i,j}$ is the allele count (genotype) of individual *i* and variant *j*, and $\hat{f_{j}}$ is the estimated allele frequency of variant *j* ($2\hat{f_{j}}$ is the mean allele count of variant *j*). Then, UΔ is the first *K* PC scores and *V* is the first *K* PC loadings, where *K* is the number of PCs computed (e.g. *K* = 20).

When there are some missing values, we compute the partial SVD similarly, except that missing values are replaced by the variant means (i.e. $G_{i,j}-2\hat{f_{j}}=0$ when $G_{i,j}$ is missing) and the $\hat{f_{j}}$ for each variant are estimated using only non-missing genotypes. Note that this decomposition is equivalent to the decomposition presented above after imputation by the variant means.

To compute this decomposition easily and efficiently, we implement an accessor that memory-map the PLINK bed file to use it directly as if it were a standard matrix. Then, we apply the same algorithm for partial SVD that is used in R packages bigstatsr and FlashPCA2, namely the implicitly restarted Arnoldi method (Abraham *et al.*, 2017; Lehoucq and Sorensen, 1996; Privé *et al.*, 2018). This algorithm, implemented in R package RSpectra, requires a function that computes the matrix-vector multiplication of the scaled genotype matrix with a given vector. We implement such multiplication in parallel from a PLINK bed file.

### 3.2 Robust Mahalanobis distance

Mahalanobis distances are computed as $d{\left(x\right)}^{2}={\left(x-\mu \right)}^{T}\Sigma^{-1}(x-\mu ),$ where *μ* and Σ are (robust) estimators of location and covariance. We use these distances for many applications in this paper. When *x* is multivariate Gaussian data with *K* dimensions, the squared distances follow a $\chi^{2}(K)$ distribution. If *x* represents PC scores of centered data and if we use standard estimates, then *μ* = 0 and $\Sigma =I_{K}$. Yet, here we use the pairwise orthogonalized Gnanadesikan–Kettenrin robust estimates of these parameters (Gnanadesikan and Kettenring, 1972; Maronna and Zamar, 2002; Yohai and Zamar, 1988). We implement the estimation of these robust parameters in function covrob_ogk of R package bigutilsr, and the direct computation of these robust distances in function dist_ogk.

### 3.3 Detecting LD structure in PCA

For detecting outlier variants in PCA that are due to long-range LD regions, we use a similar procedure as described by Privé *et al.* (2018). Note that this procedure does not require removing any known long-range LD region *a priori*. We first apply a first round of clumping at e.g. *r*^2^>0.2, prioritizing variants by higher minor allele count. Then, we compute *K* PC scores and loadings (Section 3.1). To summarize the contribution of each variant in all *K* PC loadings, we compute the robust Mahalanobis distances of these PC loadings (Section 3.2). To capture consecutive outliers that correspond to long-range LD regions, we apply a Gaussian smoothing to these statistics (moving average with a Gaussian filter over a window with a radius of 50 variants by default).

Finally, to choose the threshold on the previously described statistics above which variants are considered outliers, we use a modified version of Tukey’s rule, a standard rule for detecting outliers (Tukey, 1977). The standard upper limit defined by Tukey’s rule is $q_{75\%}(x)+1.5\cdot \mathrm{IQR}(x)$, where *x* is the vector of computed statistics and $\mathrm{IQR}(x)=q_{75\%}(x)-q_{25\%}(x)$ is the interquartile range. One assumption of Tukey’s rule is that the sample is normally distributed; we account for skewness in the data using the medcouple as implemented in function adjboxStats of R package robustbase (Brys *et al.*, 2004; Hubert and Vandervieren, 2008). Standard Tukey’s rule also uses a fixed coefficient (1.5) that does not account for multiple testing, which means that there are always some outliers detected when using 1.5 for large samples. To solve these two potential issues, we implement tukey_mc_up in R package bigutilsr and use it here, which accounts for both skewness and multiple testing by default.

We remove the detected outlier variants, compute the PC scores and loadings again, and iterate until there is no detected outlier variant anymore. This procedure is implemented in function bed_autoSVD of R package bigsnpr.

### 3.4 Detecting outlier samples in PCA

For detecting outlier samples in PCA, we use a modified version of the Probabilistic Local Outlier Factor statistic on PCs (Kriegel *et al.*, 2009). Using K nearest neighbors (KNN), this consists in comparing the distance from a point *j* to its KNNs ($pd_{j}=\sqrt{\frac{1}{K}\sum_{k=1}^{K}d_{j↔j_{k}}^{2}}$, where *j_k_* is the *k*-th NN of *j*) with the distances from its KNNs to their respective KNNs $\left(\frac{1}{K}\sum_{k=1}^{K}pd_{j_{k}}\right)$. Intuitively, an outlier should be far from all other points, and is even more outlier if its KNNs are in a very dense cluster. Here, we use $pd_{j}/\sqrt{\frac{1}{K}\sum_{k=1}^{K}pd_{j_{k}}}$ as statistic to detect individual outliers in PCA. Note that the square root, as it otherwise detects as outlier any point that is next to a very dense cluster. We implement (the two parts of) this statistic in function prob_dist of R package bigutilsr. To make it fast, we use the fast KNN implementation of R package nabor (Elseberg *et al.*, 2012) and parallelize it in function knn_parallel of package bigutilsr. Automatic outlier detection is difficult; therefore, we recommend users to choose a threshold for this statistic to define outliers based on visual inspection (using the histogram of these statistics and the PC scores colored by these statistics; see e.g. Fig. 2).

As for detecting samples that have a different ancestry from most of the samples in the data, i.e. for restricting to homogeneous samples, we compute the pairwise orthogonalized Gnanadesikan–Kettenrin robust Mahalanobis distances on PC scores (Section 3.2). We then restrict to individuals whose log-distance (alternatively *P*-value) is smaller (larger) than some threshold determined based on visual inspection.

### 3.5 Projecting PCs from a reference dataset

To project a target genotype dataset to the PCA space from a reference dataset (e.g. the 1000 Genomes data), we implement the following three steps in function bed_projectPCA of package bigsnpr: (i) matching the variants of each dataset, including removing ambiguous alleles [A/T] and [C/G], and matching strand and direction of the alleles; (ii) computing PCA of the reference dataset using the matched variants only and (iii) projecting computed PCs to the target data using an optimized implementation (see Supplementary Material) of the Online Augmentation, Decomposition, and Procrustes (OADP) transformation (Zhang *et al.*, 2020). To project individuals from the same dataset as the individuals used for computing PCA, we provide function bed_projectSelfPCA. Note that the new individuals to be projected should not be related to the ones used for computing PCA (cf. Section 4.3).

### 3.6 Data

We provide and use a subsetted version of the 1000 Genomes (1000G) project data (1000 Genomes Project Consortium *et al.*, 2015; Meyer, 2019). Variants are restricted to the ones in common with HapMap3 or UK Biobank (Bycroft *et al.*, 2018; International HapMap 3 Consortium *et al.*, 2010). Moreover, we apply some quality control filters; we remove variants having a minor allele frequency <0.01, variants with *P*-value of the Hardy–Weinberg exact test <$10^{-50}$, and non-autosomal variants. To remove related individuals with second-degree relationship or more, we apply KING-relatedness cutoff of 0.0884 to the data using PLINK 2.0 (Chang *et al.*, 2015; Manichaikul *et al.*, 2010). This results in 2490 individuals and 1 664 852 variants of the 1000G project (phase 3) in PLINK bed/bim/fam format. Resulting PLINK files and R code to generate these files are made available at https://doi.org/10.6084/m9.figshare.9208979.v3. To easily download this data, we provide function download_1000G in R package bigsnpr.

In this paper, we also analyze the UK Biobank data (https://www.ukbiobank.ac.uk/). We apply some quality control filters; we remove individuals with >10% missing values, variants with >1% missing values, variants having a minor allele frequency <0.01, variants with *P*-value of the Hardy–Weinberg exact test <$10^{-50}$ and non-autosomal variants. This results in 488 371 individuals and 504 139 variants. When removing related individuals, we use the list of individual pairs reported by the UK Biobank.

## 4 Results

### 4.1 Application to the UK Biobank

To demonstrate that we provide very fast implementations of the different methods presented in this paper, we apply them to the UK Biobank (UKBB). We use 20 physical cores for most of the computations [CPU: Intel(R) Xeon(R) Silver 4114, 2.20 GHz]. It takes 22 min to perform a first phase of clumping on 406 545 unrelated individuals genotyped over 504 139 variants, which reduces the number of variants to 261 307. It then takes 34 min to compute the first 20 PCs using these 261 307 variants. When performing the automatic procedure for LD detection, it takes 5 h to perform the initial clumping step, 6 rounds of computation of PCs and 5 rounds of outlier variant detection (i.e. five iterations of outlier detection and one final computation of PCs).

**Fig. 1.**
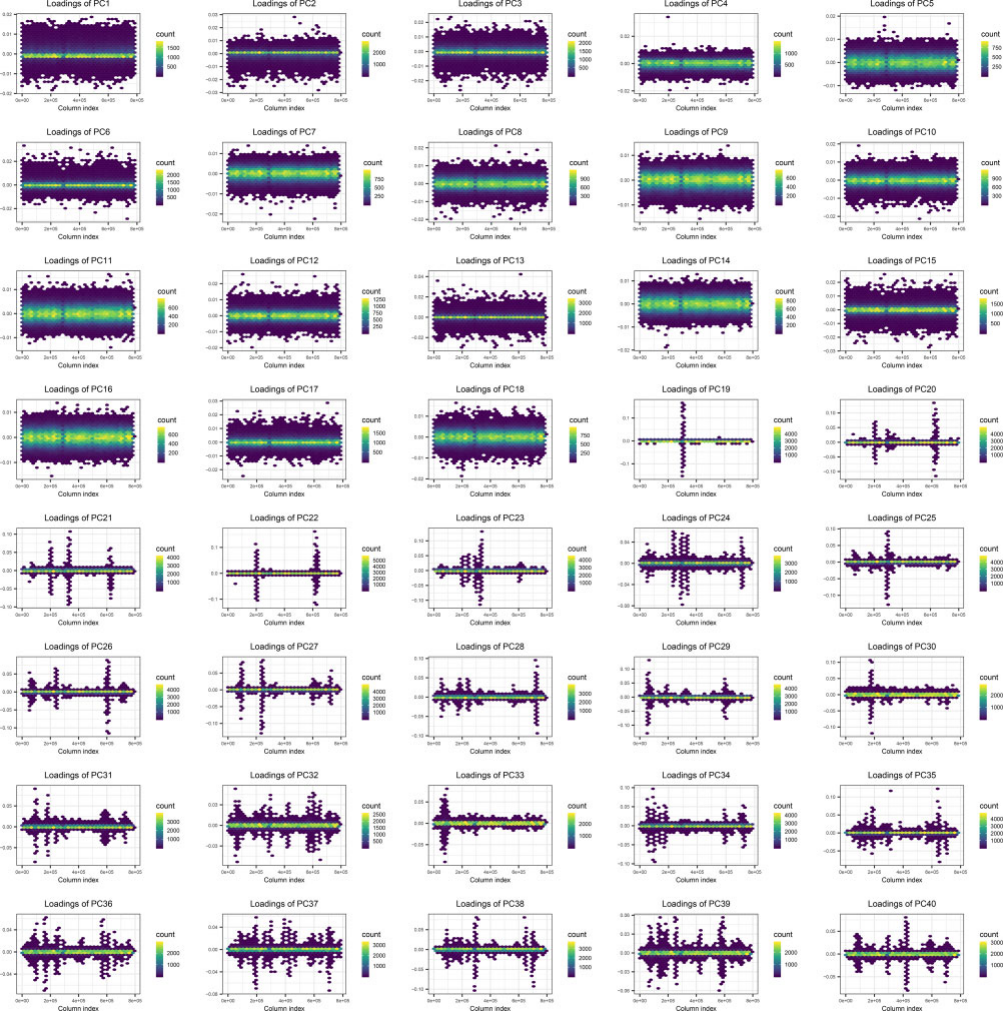
PC loadings 1–40 reported by the UK Biobank. Column indices of variants in the data, ordered by chromosome and physical position, are represented on the *x*-axis, and the value of loadings is represented on the *y*-axis. Points are hex-binned. (**a**) Distribution of statistics (S). (**b**) PC scores 13–20 of 1000G, colored by the statistic (S) used to define outliers. A few points with higher values for this statistic S appear as outliers in PC17–PC20. (**c**) PC scores 13–20 of 1000G, colored by being detected as an outlier. Threshold of being an outlier is determined based on histogram (a) (Color version of this figure is available at *Bioinformatics* online.)

When applying our automatic procedure to remove long-range LD regions, it does not converge after five iterations for the UK Biobank, i.e. it keeps detecting long-range LD regions at each iteration (represented by peaks in PC loadings). Therefore, we are able to capture only 16 PCs that show stratification that is not LD structure (Supplementary Figs S8–S10). Similarly, PC loadings reported by the UK Biobank clearly show that PC19–PC40 capture LD structure, which is also the case for PC16 and PC18, although less pronounced (see peaks in Fig. 1). These include e.g. one region on chromosome 6 (70–91 Mbp) that is captured in PC19 (Supplementary Fig. S10) and that was not previously reported in Price *et al.* (2008).

As for other analyses, it takes 8 min to match the 1000G data to the UKBB data and compute 20 PCs of the 1000G data using the automatic LD detection technique. It takes 12 min more to perform the OADP projection of all 488 371 UKBB individuals onto the PCA space computed using the 1000G data. Finally, it takes only 6 min to compute the 30-nearest neighbors of 20 PC scores for 406 545 UK Biobank individuals, which is the most computationally demanding step when computing the statistics used to detect individual outlier samples (Section 3.4).

### 4.2 Outlier sample detection

**Fig. 2.**
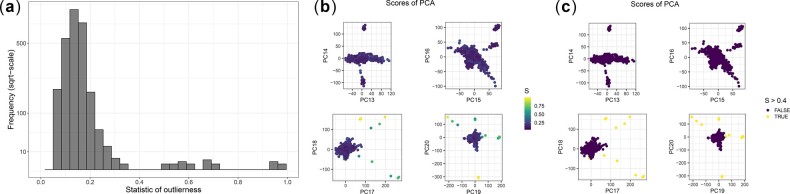
Outlier detection in the 1000 Genomes (1000G) project, using prob_dist (Section 3.4)

To detect a few outlier samples, we compare the standard rule of ‘6 SDs from the mean’ (6SD) used in e.g. EIGENSOFT to the statistic we propose in Section 3.4. Our statistic identifies only isolated samples or isolated pairs that seems to be outliers driving structure of PC17–PC20 of 1000G (Fig. 2). All but one outlier are distantly related pairs that disappear if using a more stringent threshold on relatedness (i.e. using a KING-relatedness cutoff of ∼0.0442 instead of ∼0.0884, see tutorial in section ‘code availability’). In contrast, rule 6SD identifies a lot of outliers, of which some are part of a relatively large cluster (Supplementary Fig. S1). We recall that, if all PCs are normally distributed, after correcting for multiple testing of 2500 individuals and 20 PCs, the probability of detecting one outlier or more using 6SD is only of 0.0001.

As for restricting to homogeneous samples, we compare the use of the robust Mahalanobis distance we propose here to the use of R package aberrant, which was used to report the homogeneous ‘White British’ subset in the UKBB (Bellenguez *et al.*, 2012; Bycroft *et al.*, 2018). We visually choose a threshold of 5 on the log-distance and show that this gives a similar subset of individuals than the ‘White British’ subset reported by the UK Biobank (Supplementary Fig. S2). Moreover, when using this threshold, only 3 out of 10 936 people of self-reported Asian ancestry (1 ‘Chinese’ and 2 ‘Indian’) are kept, and 1 ‘African’ out of 7622 people with Black background is kept (Supplementary Table S1). In contrast, 416 492 out of 431 090 ‘British’ (96.6%) and 12 620 out of 12 759 ‘Irish’ (98.9%) are kept. Results are very similar to the set of ‘White British’ made using R package aberrant (Supplementary Fig. S2).

### 4.3 Projecting onto the PCA space from a reference dataset

**Fig. 3.**
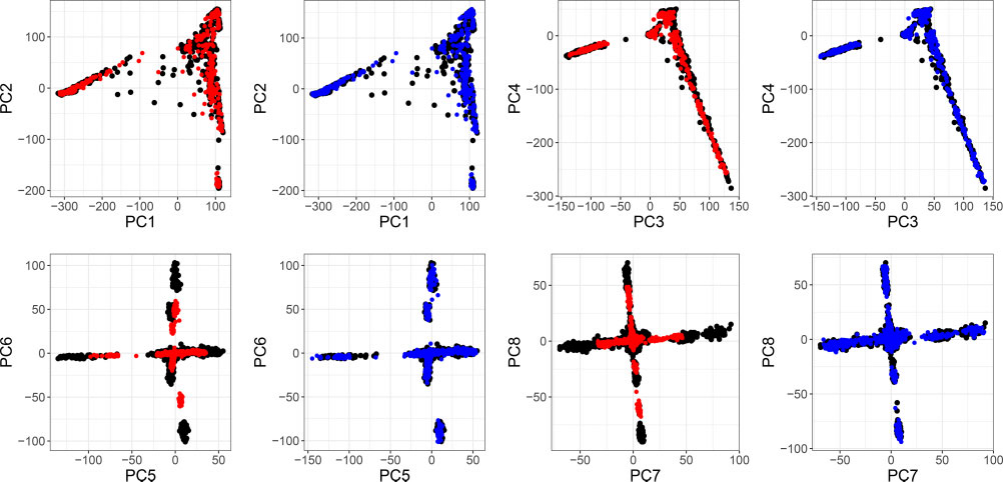
PC scores 1–8 of the 1000 Genomes project. Black points are the 60% individuals used for computing PCA. Red points are the 40% remaining individuals, projected by simply multiplying their genotypes by the corresponding PC loadings. Blue points are the 40% remaining individuals, projected using the OADP transformation. Estimated shrinkage coefficients for these eight PCs are 1.01 (PC1), 1.02, 1.06, 1.09, 1.50 (PC5), 1.69, 1.98 and 1.39. (Color version of this figure is available at *Bioinformatics* online.)

We use 60% of individuals in the 1000G data (Section 3.6) to compute *K* = 20 PCs. Then, we project the remaining 40% individuals using three methods: 1/ simply multiplying the genotypes of these individuals by the previously computed loadings; 2/ correcting the simple projections using asymptotic shrinkage factors as determined by R package hdpca v1.1.3 (Dey and Lee, 2019), with all eigenvalues derived from the genetic relationship matrix computed with bed_tcrossprodSelf, one of the new functions of R package bigsnpr; and 3/ the OADP projection (Section 3.5). When simply projecting using loadings, there is negligible shrinkage for PC1 and PC2, a small shrinkage for PC3 and PC4 and a large shrinkage for PC5–PC8 (Fig. 3). In contrast, there is no visible shrinkage when projecting new individuals with OADP (Fig. 3). Simple projection is affected even more by this shrinkage for PC9–PC20, while OADP still appears free of this bias (Supplementary Fig. S3). We show the same results when projecting the full UK Biobank data onto PCA computed using 1000G data (Supplementary Fig. S6). When correcting projected PC scores with asymptotic shrinkage factors, bias is smaller than with simple projection, yet, there is a visible bias for PC7–PC8 (Supplementary Fig. S4). Finally, to assess if OADP could be used to project individuals that are related to some individuals that were used to compute PCA, we projected these 60% individuals (as if we were projecting their monozygotic twins) using OADP. Projections of related individuals using OADP suffers from some bias in reverse direction (Supplementary Fig. S5).

**Fig. 4.**
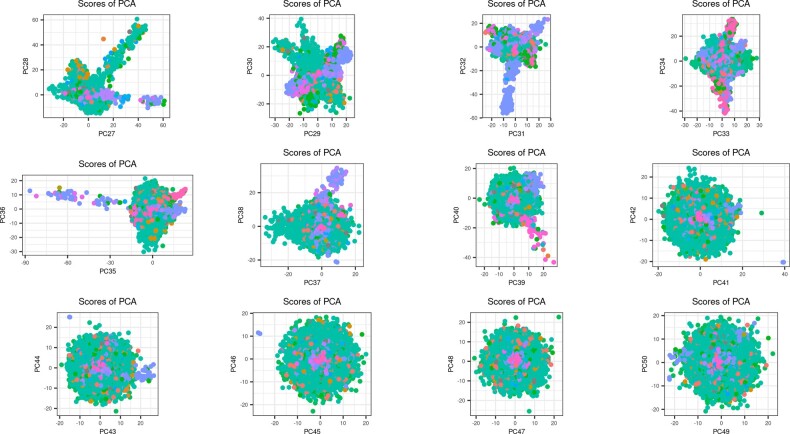
PC scores 27–50 computed on the UK Biobank using 48 942 individuals of diverse ancestries. These individuals are the ones resulting from removing all related individuals and randomly subsampling the British and Irish individuals. Different colors represent different self-reported ancestries. (Color version of this figure is available at *Bioinformatics* online.)

**Fig. 5.**
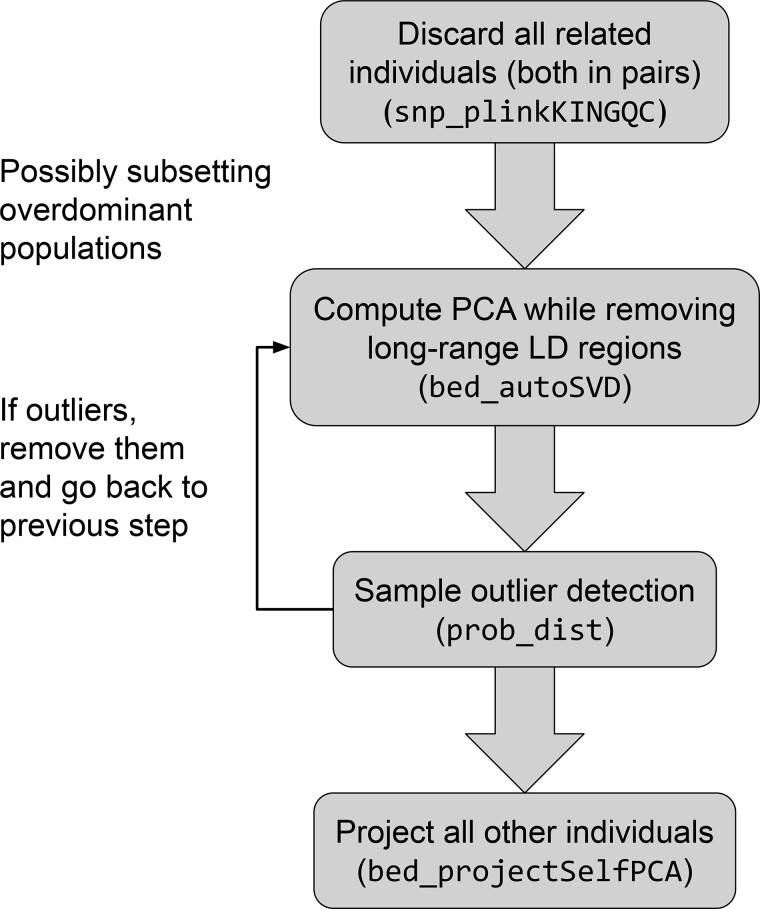
Proposed pipeline for computing PCs using R packages bigsnpr and bigutilsr

When computing the PCs on the UK Biobank using 406 545 unrelated individuals and 171 977 variants, and projecting the 1000G data onto this reference PCA space, shrinkage is much smaller (≤1.08 for all 20 first PCs, Supplementary Fig. S7). Overall, this shrinkage for simple projection decreases with an increased sample size (Table 2).

**Table 2.** Shrinkage coefficients when projecting new individuals onto reference PCA space

Dataset	Sample size (×1000)	Number of variants (×1000, after LD removal)	Shrinkage (PC 1–5–10–20)
1000G	1.5	393	1.01–1.50–3.14–6.70
1000G	2.5	229	1.01–1.36–2.84–6.75
UKBB	49.0	282	1.00–1.04–1.12–1.43
UKBB	406.5	172	1.00–1.01–1.04–1.08

*Note*: We list the dataset, the sample size and number of variants used to compute the final PCA. As expected, the shrinkage bias only becomes negligible if the PCA is conducted on large samples.

### 4.4 Capturing subtle population structure in the UK Biobank

We recomputed PCA in the UK Biobank after restricting the individuals included in the computations: we randomly subsampled UKBB data to use only 10 000 British individuals (out of 431 029) and 5000 Irish individuals (out of 12 755), while keeping all individuals with other or unknown self-reported ancestry. We further removed all pairs of related individuals reported by the UKBB (i.e. both individuals in each pair). This resulted in 48 942 individuals that we used to compute 50 PCs, which took <3 h using function bed_autoSVD (that converged after four iterations of automatic LD removal). We show that we are able to capture more PCs (at least 40 instead of 16–18) that display visual population structure (Fig. 4 and Supplementary Fig. S12). We then projected all 439 429 remaining individuals from UKBB onto this PCA space in 21 min only using our implementation of the OADP projection (function bed_projectSelfPCA). Note that these individuals should not be related to any of the 48 942 individuals used for training PCA because we removed both individuals from each pair of related individuals in the UKBB. Projection of new individuals show again a clear shrinkage when using simple projection (between 1.00 for PC1 and 1.80 for PC50), but no visible bias when using OADP projection (Supplementary Fig. S13).

### 4.5 PCA and missing value imputation

As we compute PCA on data with missing values, although we restrict to variants with <1% missing values, we analyze hereinafter the effect of imputation of missing values before computing PCA. We compare four different imputation methods and two different sets of individuals. In the UK Biobank imputed data, ∼1000 individuals have been removed because of a high number of missing values or a high heterozygosity, as compared to the genotyped data (Bycroft *et al.*, 2018). When computing PCA with mean imputation and using all genotyped individuals, PC16 captures individuals with very high heterozygosity (Supplementary Fig. S11). When restricting to imputed individuals only, i.e. after removing individuals with very high heterozygosity, PC16 completely disappears and new PC16–PC19 correspond to previous PC17–PC20 (Supplementary Table S2). When using dosage data instead of genotype data with mean imputation, PCA is globally unchanged (Supplementary Table S3). Overall, if we choose to use either one of the following four imputation methods: mean imputation, random imputation according to allele frequencies, using reported dosage data from BGEN files, or imputation of genotyped data based on machine learning using function snp_fastImpute of R package bigsnpr (Privé *et al.*, 2018), resulting PCs are always very similar (absolute correlation larger than 0.99 for the 20 computed PCs; results partially shown in Supplementary Tables S2 and S3). This justifies performing PCA with mean imputation directly on PLINK bed files with a few missing values; this has the advantage to be much faster than having to impute genotyped data using snp_fastImpute, which took 4 days for 406 545 individuals and 240 444 variants, or based on external reference datasets.

## 5 Discussion

In this work, we have compiled different pitfalls that can arise with PCA of genetic data. Then, we have investigated possible solutions to these pitfalls and selected the ones that we found most advantageous, both with respect to properties such as accuracy and robustness, but also computational efficiency and ease of use. We then implemented these solutions in R packages bigsnpr and bigutilsr. The new functions we provide in R package bigsnpr can be directly applied to genotypes stored as PLINK bed/bim/fam files with some missing values. This contrasts with previous releases of package bigsnpr that could only use format ‘bigSNP’. This data format can store both genotype calls and dosages, but requires conversion from other formats and imputation of missing values using functions provided in the package (Privé *et al.*, 2018). As PCA is a useful tool on its own and does not require extensive imputed data, we therefore decided that operating directly on PLINK files with a few missing values would be more practical for users.

We summarize our work into several recommendations for computing PCA, and propose the pipeline shown in Figure 5. Note that we have not included standard steps such as initial quality control filters and post-analysis checks (e.g. visual inspection of different plots). This pipeline requires removing all related individuals, for which we provide an R wrapper to PLINK’s implementation of KING robust kinship coefficients (Chang *et al.*, 2015; Manichaikul *et al.*, 2010). Note that one should remove both individuals in each pair of related individuals. This ensures that the projected individuals are not related to the ones used for computing PCA, since we showed that relatedness is a problem when using the OADP projection (Supplementary Fig. S5). After selecting a subset of individuals, we apply several steps of outlier detection, one for outlier variants that capture long-range LD variation (automatic), and one for detecting outlier samples (semi-automatic and visual). To make these steps more computationally efficient, we explored solutions for not recomputing PCA from scratch when removing a few samples or a few variants. Using educated guesses in R package PRIMME based on low-rank approximations of the updated PCA seemed to be a promising approach but did not reduce computation time by much, so we did not pursue this idea (Brand, 2003; Wu *et al.*, 2017).

Once PCA is done, one should check the PC scores (scores of each individual for each PCA dimension) and PC loadings (weights for each variant for each PCA dimension). We differentiate PCs in three broad types: the ones capturing LD structure, the ones capturing population structure and noise. We expect population structure to be evenly distributed along the genome so that loadings are normally distributed around 0 (with small effect sizes). In contrast, long-range LD structure is essentially capturing the variation inside one long-range LD region (so localized in the genome), so that we expect the loadings to be very large in that region only (one peak). Therefore, PCs capturing LD structure can be identified by looking for peaks in PC loadings (e.g. PC17–PC20 in Supplementary Fig. S10). To identify which PCs capture population structure, and which ones are probably just noise, one should also look at PC scores (colored by ancestry if possible). PCs with no visible population stratification, i.e. where all individuals are normally distributed around 0, can be considered as noise. As in many applications, we believe a compromise between signal and noise should be preferred. Therefore, we recommend using only PCs that show structure (e.g. PC1–PC16 in Supplementary Fig. S9) and excluding PCs that do not seem to capture any population structure (e.g. PC17–PC20 in Supplementary Fig. S9).

When analyzing a dataset that is composed mainly of one population (e.g. British people in the UK Biobank), we found that it is useful to subset these individuals to reduce the imbalance between the different population sizes. Likewise, previous works have shown that uneven population sizes can distort PCs (McVean, 2009; Novembre and Stephens, 2008). Indeed, when subsetting British and Irish people in the UK Biobank data, we are able to capture a lot more PCs that show population structure with <50 K individuals compared to when using >400 K individuals who are mostly composed of British and Irish people. Determining how much overdominant populations should be subsampled to maximize population structure captured by PCA is a direction of future work. The remaining individuals can then be projected onto the resulting PCA space using the OADP projection we recommend in this paper. This suggests that designs such as the 1000 Genomes project, which gathered around 100 people for each of 26 different populations, are highly relevant for capturing population structure (1000 Genomes Project Consortium *et al.*, 2015).

In contrast, a common strategy in genetic analyses is to restrict the analysis to a homogeneous sample to reduce risk of confounding due to population stratification. For that purpose, we show that using the Mahalanobis distance on PC scores can efficiently achieve this goal, which we used in previous analyses (Privé *et al.*, 2019). When the homogeneous sample is not predominant in the dataset, one solution is to compute the center and covariance of the robust Mahalanobis distance using only the population of interest, and then computing the distances for all individuals using these robust estimates.

The ubiquitous use of PCA in a wide variety of genomic analyses makes it difficult to establish universal guidelines for such analysis. Although we have tackled many problems related to computing PCA on genotype data in this paper, we do not answer other important problems, such as how to best control for population structure in genomic analyses. For example, when conducting a GWAS, should one restrict to a homogenous sample, or is it enough to just include PCs that capture population structure as covariates, or should one also use PCs as covariates in mixed linear models (Loh *et al.*, 2015b; Price *et al.*, 2010)? Similarly, in some analyses, it may be beneficial to include PCs that capture long-range or even inter-chromosomal LD. More work is needed to understand these fundamental problems, and to provide precise guidelines for conducting successful GWAS, heritability and other genomic analyses where PCA is used. These are directions of future work.

Finally, although we have focused on PCA of genotype data in this paper, we believe most of the results presented here are not inherent to genotype data, and can be transferred to e.g. other omics data as well. For example, PCs can be used to account for confounding in other data as well (Pickrell *et al.*, 2010). Then, outlier and homogeneous sample detection can be used on PCs of other types of data. Moreover, projection of scores will also be a problem for other omics data where the number of variables used is larger than the number of samples used for computing PCA. Finally, using ‘populations’ with approximately the same size is relevant for other biological data as well. However, other pitfalls might apply when using other types of data; e.g. methylation data can be confounded by factors such as age and sex, and it might be beneficial to remove the methylation probes that are associated with these confounding factors before computing PCA (Decamps *et al.*, 2020).

## Supplementary Material

btaa520_supplementary_data
