# Supplementary material for: Efficient toolkit implementing best practices for principal component analysis of population genetic data
Source: Bioinformatics. 2020 May 16;36(16):4449–57. doi: 10.1093/bioinformatics/btaa520 (PMC7750941; doi:10.1093/bioinformatics/btaa520)
Supplement: btaa520_supplementary_data [file btaa520_supplementary_data.pdf]

## Optimised OADP transformation

We implement an optimised version of the Online Augmentation, Decomposition, and Procrustes (OADP) transformation when using $K'' = K' = K$ (Zhang *et al.* 2020). We assume that the $K$-partial Singular Value Decomposition (SVD) of the scaled reference matrix $X$ (of size $n \times p$) has been computed as $U\Delta V^T$. There are several steps to perform OADP transformation for each sample $y$ (of size $1 \times p$) of target matrix $Y$ (of size $m \times p$):

1. Calculate $l = y \cdot V$ (of size $1 \times K$), where $V$ are the K PC loadings. And $g = y \cdot h^T$ (of size $1 \times 1$), where $h = (y - l \cdot V^T)/||y - l \cdot V^T||_2$. Actually, $||y - l \cdot V^T||_2^2 = y \cdot y^T - 2 \cdot y \cdot V \cdot l^T + l \cdot V^T \cdot V \cdot l^T = y \cdot y^T - l \cdot l^T$ and $y \cdot (y - l \cdot V^T)^T = y \cdot y^T - y \cdot V \cdot l^T = y \cdot y^T - l \cdot l^T$. Then $g = \sqrt{y \cdot y^T - l \cdot l^T}$.

2. Calculate $Q^T Q$ where
$$Q = \begin{bmatrix} \Delta & l^T \\ 0 & g \end{bmatrix}$$
so that
$$Q^T Q = \begin{bmatrix} \Delta^2 & \Delta \cdot l^T \\ l \cdot \Delta & g^2 + l \cdot l^T \end{bmatrix} = \begin{bmatrix} \Delta^2 & \Delta \cdot l^T \\ l \cdot \Delta & y \cdot y^T \end{bmatrix}.$$
Note that we do not actually need to compute $g$, and that we can update only the last row and column of $Q^T Q$ instead of computing it from an updated version of $Q$.

3. Get the eigen decomposition $Q^T Q = V' \Delta'^2 V'^T$ (truncated to $K$ components out of the $K + 1$). Let us denote $V_2 = V' \Delta'$.

4. Calculate
$$\begin{bmatrix} \widetilde{U} \\ \widetilde{u} \end{bmatrix} = \begin{bmatrix} U & 0 \\ 0 & 1 \end{bmatrix} V_2 = \begin{bmatrix} U V_2[1{:}K,\ ] \\ V_2[K{+}1,\ ] \end{bmatrix}$$

5. Find the Procrustes transformation from $\widetilde{U}$ to $U\Delta$. As $\widetilde{U}$ and $U\Delta$ have both their columns centered already (since $U$ does), the Procrustes transformation $\rho \widetilde{U} A$, where $\rho$ is a scaling coefficient and $A$ is an orthonomal projection matrix that minimise the Frobenius norm of $(\rho \widetilde{U} A - U)$, is given by $A = V'' U''^T$ and $\rho = \frac{\text{trace}(\Delta'')}{\text{trace}(\widetilde{U}^T \widetilde{U})}$ where $U'' \Delta'' V''^T$ is the full SVD of $(U\Delta)^T \widetilde{U}$ (Wang *et al.* 2015). As $U^T U = I$, we note that $(U\Delta)^T \widetilde{U} = \Delta V_2[1{:}K,\ ]$ and that $\rho = \frac{\text{trace}(\Delta'')}{\text{trace}(V_2[1{:}K,\ ]^T V_2[1{:}K,\ ])}$, therefore we do not need to explicitly compute $\widetilde{U}$ and do not need $U$.

6. Apply the previous transformation to $\widetilde{u}$ to get the projection of $y$ in the reference PCA space (i.e. $\rho \widetilde{u} A$).

# References


Wang, C., Zhan, X., Liang, L., Abecasis, G. R., and Lin, X. (2015). Improved ancestry estimation for both genotyping and sequencing data using projection procrustes analysis and genotype imputation. *The American Journal of Human Genetics*, **96**(6), 926–937.

Zhang, D., Dey, R., and Lee, S. (2020). Fast and robust ancestry prediction using principal component analysis. *Bioinformatics*. btaa152.

# Sample outlier detection

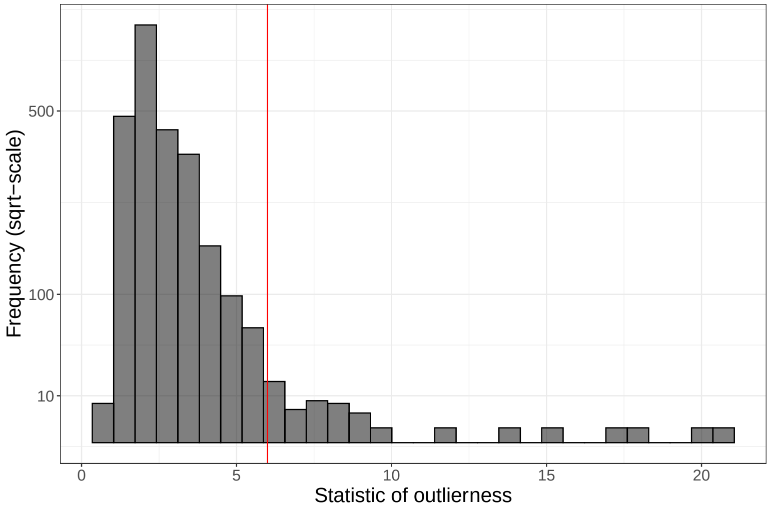


(a) Distribution of statistics (S2) and default threshold (6, in red)

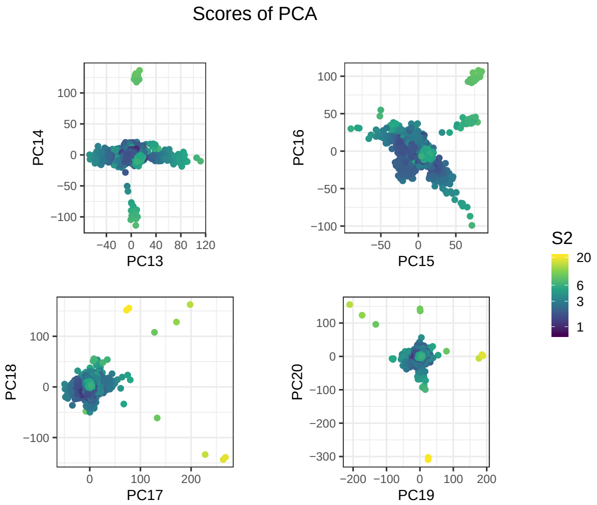


(b) Principal Component (PC) scores 13 to 20 of 1000G, colored by statistic used to detect outliers (maximum number of SDs from the mean, log-scale).

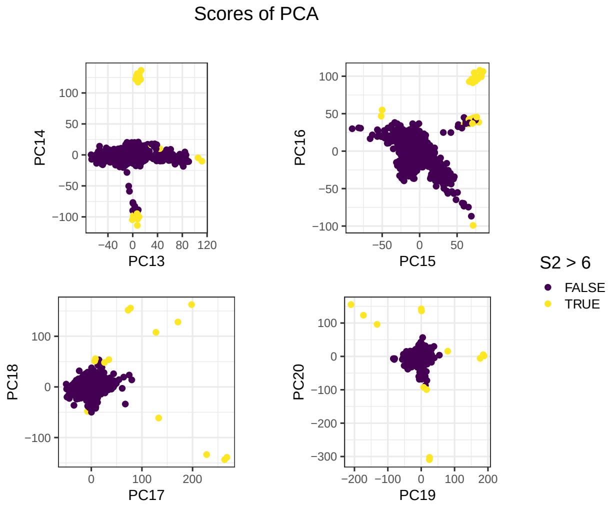


(c) Principal Component (PC) scores 13 to 20 of 1000G, colored by being detected as an outlier.

Figure S1: Outlier detection in the 1000 Genomes (1000G) project, using the rule "6 SDs from the mean", i.e. where S2 is the maximum (for all PCs) number of SDs from the mean (Section 2.4).

Table S1: Number of UKBB individuals with (log squared) Mahalanobis distance lower than some threshold (top), and grouped by self-reported ancestry (left). Note that "< 12" includes all individuals.

| | < 3 | < 4 | < 5 | < 6 | < 7 | < 8 | < 9 | < 10 | < 11 | < 12 |
|---|---|---|---|---|---|---|---|---|---|---|
| Prefer not to answer | 484 | 1013 | 1062 | 1099 | 1139 | 1177 | 1279 | 1405 | 1471 | 1583 |
| Do not know | 36 | 68 | 76 | 84 | 92 | 118 | 155 | 188 | 196 | 204 |
| White | 186 | 422 | 457 | 483 | 513 | 533 | 543 | 543 | 545 | 546 |
| Mixed | 2 | 6 | 6 | 7 | 8 | 15 | 26 | 42 | 46 | 46 |
| Asian or Asian British | | | | | | 3 | 20 | 40 | 42 | 42 |
| Black or Black British | 1 | 2 | 2 | 2 | 2 | 2 | 2 | 4 | 6 | 26 |
| Chinese | | 1 | 1 | 1 | 1 | 2 | 5 | 21 | 1423 | 1504 |
| Other ethnic group | 57 | 230 | 261 | 314 | 469 | 885 | 1939 | 2761 | 3681 | 4356 |
| British | 191713 | 400516 | 416492 | 424490 | 427769 | 429172 | 431026 | 431082 | 431089 | 431090 |
| Irish | 1416 | 12039 | 12620 | 12700 | 12734 | 12743 | 12759 | 12759 | 12759 | 12759 |
| Any other white background | 1468 | 4747 | 6953 | 9341 | 12979 | 14613 | 15741 | 15810 | 15820 | 15820 |
| White and Black Caribbean | 1 | 4 | 4 | 4 | 9 | 35 | 142 | 537 | 589 | 597 |
| White and Black African | 1 | 3 | 3 | 4 | 6 | 29 | 99 | 333 | 400 | 402 |
| White and Asian | 4 | 7 | 13 | 23 | 79 | 350 | 651 | 790 | 802 | 802 |
| Any other mixed background | 24 | 66 | 87 | 155 | 274 | 391 | 595 | 884 | 990 | 996 |
| Indian | | 2 | 2 | 5 | 6 | 29 | 1682 | 5700 | 5716 | 5716 |
| Pakistani | | | | | 1 | 13 | 532 | 1747 | 1748 | 1748 |
| Bangladeshi | | | | | | | 2 | 220 | 221 | 221 |
| Any other Asian background | | | | 1 | 6 | 66 | 427 | 1364 | 1730 | 1747 |
| Caribbean | | | | | | | 3 | 113 | 1323 | 4299 |
| African | | 1 | 1 | 1 | 1 | 1 | 3 | 58 | 350 | 3205 |
| Any other Black background | | | | | | 1 | 3 | 22 | 49 | 118 |
| All | 195393 | 419127 | 438040 | 448714 | 456088 | 460178 | 467634 | 476423 | 480996 | 487827 |

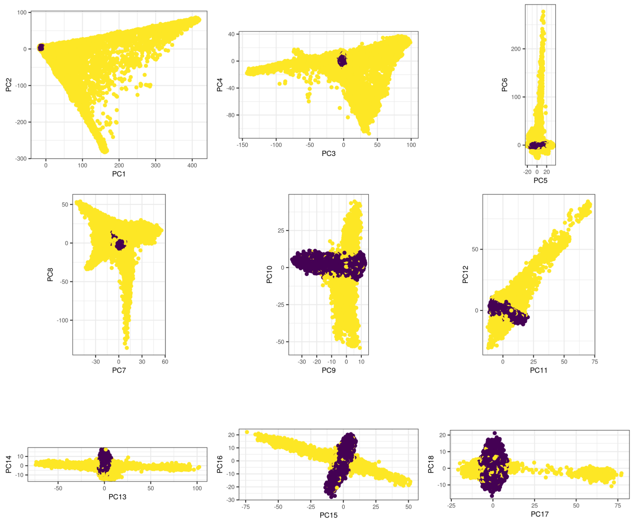


(a) PCs 1 to 20 of UKBB, colored by whether it is in the group of “White British” reported by the UKBB (blue).

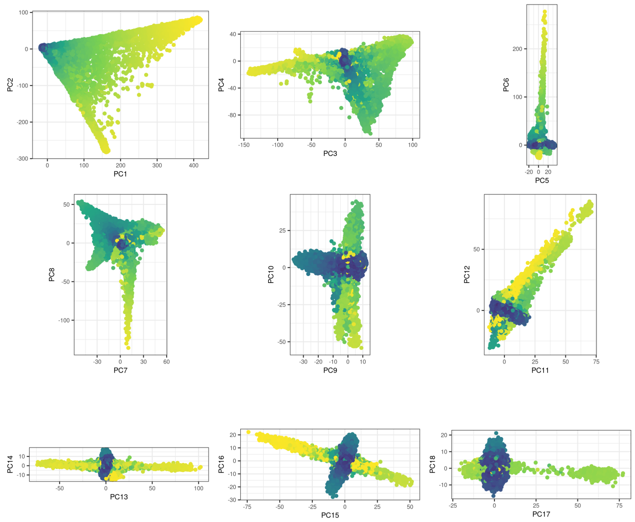


(b) PCs 1 to 20 of UKBB, colored by robust Mahalanobis distances computed on PCs (log-scale).

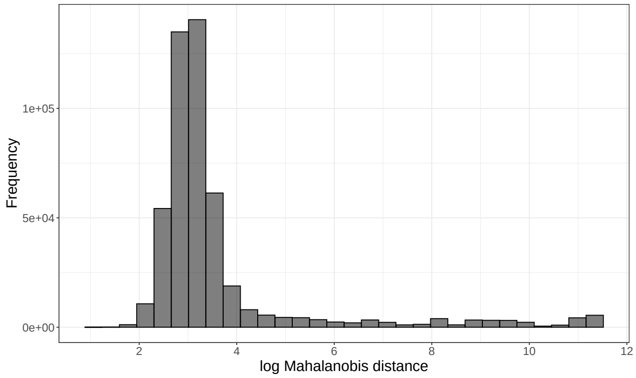


(c) Distribution of (log squared) distances.

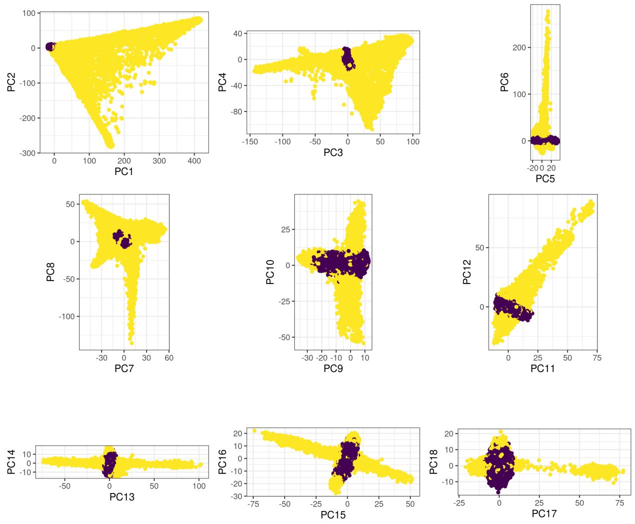


(d) PCs 1 to 20 of UKBB, colored by being detected as an outlier. Threshold of being considered as an outlier is determined based on histogram (c), where the threshold of 5 is chosen for the logarithm of the distances.

).

Figure S2: Homogeneous sample detection in the UK Biobank (UKBB), using robust Mahalanobis distances computed on the first 20 Principal Component scores (PCs) of UKBB.).

## Projection onto reference PCA space

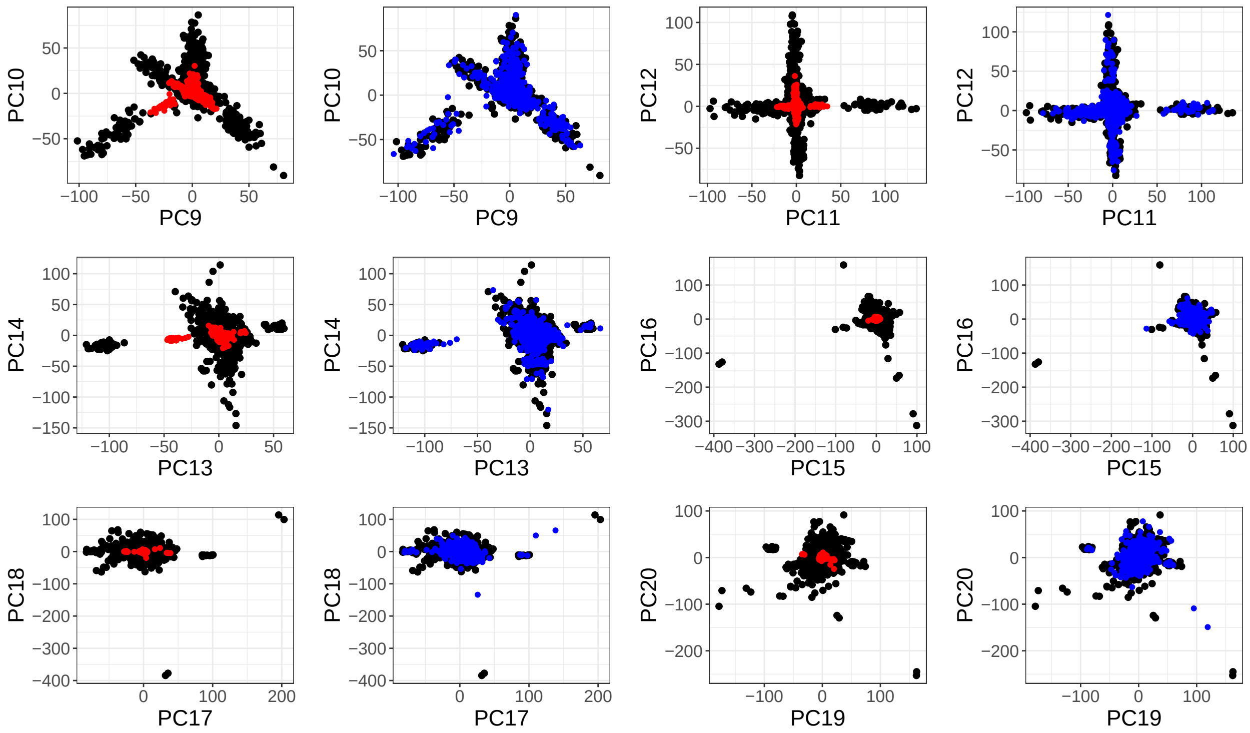


Figure S3: Principal Component (PC) scores 9 to 20 of the 1000 Genomes project. Black points are the 60% individuals used for computing PCA. Red points are the 40% remaining individuals, projected by simply multiplying their genotypes by the corresponding PC loadings. Blue points are the 40% remaining individuals, projected using the Online Augmentation, Decomposition, and Procrustes (OADP) transformation. Estimated shrinkage coefficients (comparing red and blue points) for these PCs are 2.79, 3.14 (PC10), 3.64, 3.18, 2.47, 3.88, 5.31, 5.84, 3.45, 6.55, 3.68 and 6.70 (PC20).

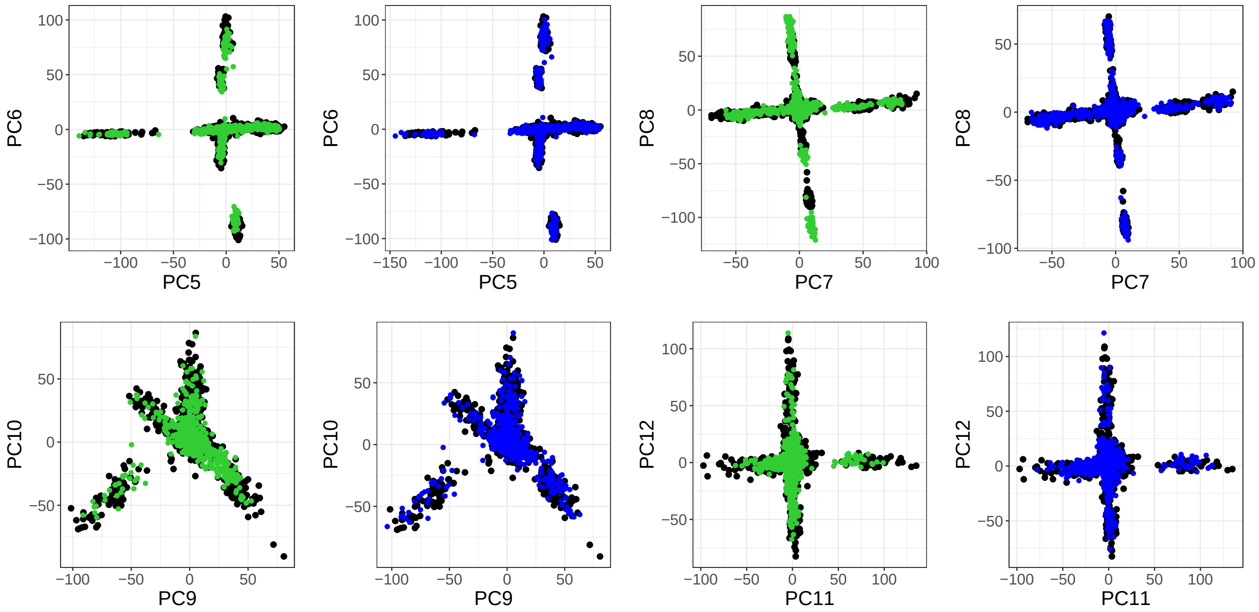


Figure S4: Principal Component (PC) scores 5 to 12 of the 1000 Genomes project. Black points are the 60% individuals used for computing PCA. Green points are the 40% remaining individuals, projected by multiplying their genotypes by the corresponding PC loadings, further corrected using theoritical asymptotic shrinkage factors (values for the first 12 PCs: 1.01 (PC1), 1.02, 1.07, 1.10, 1.43 (PC5), 1.54, 1.74, 1.79, 2.47, 2.77 (PC10), 2.84 and 3.15). Blue points are the 40% remaining individuals, projected using the Online Augmentation, Decomposition, and Procrustes (OADP) transformation.

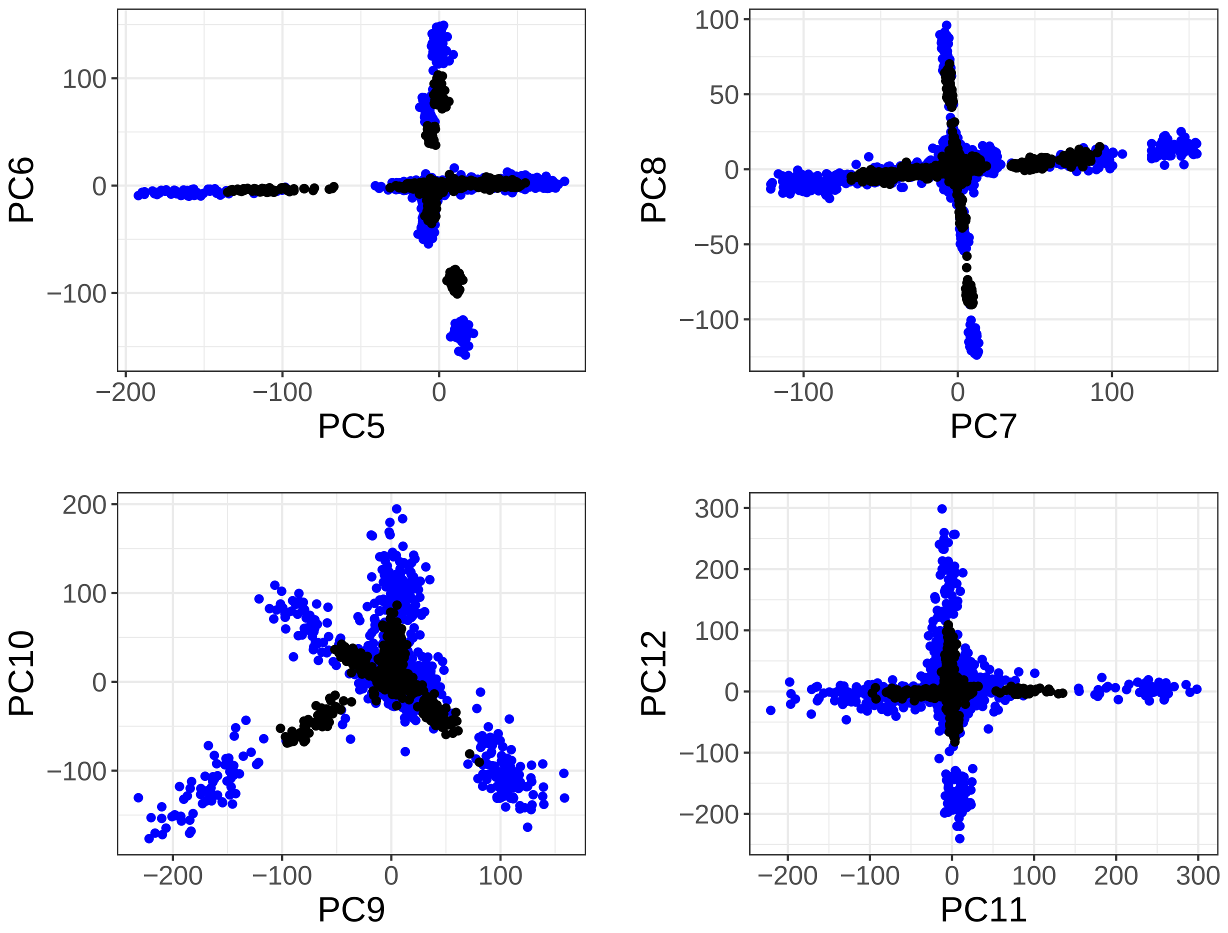


Figure S5: Principal Component (PC) scores 5 to 12 of the 1000 Genomes project. Black points are the 60% individuals used for computing PCA. Blue points are the same 60% individuals, projected using the Online Augmentation, Decomposition, and Procrustes (OADP) transformation.

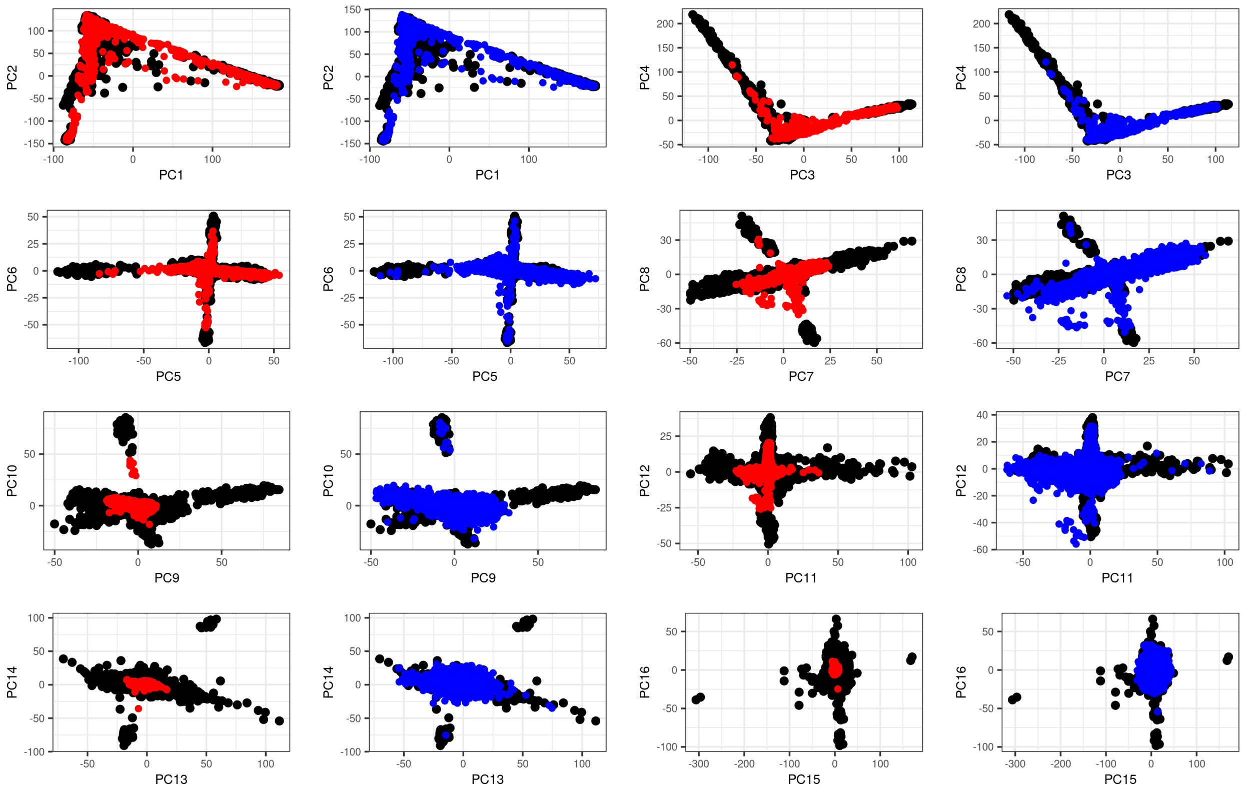

Figure S6: Principal Component (PC) scores 1 to 16 of the 1000 Genomes project and projected individuals from the UK Biobank. Black points are PC scores of 1000G individuals used for computing PCA. Red points are the individuals from UKBB, projected by simply multiplying their genotypes by the corresponding PC loadings. Blue points are the 488,371 individuals from the UK Biobank, projected using the Online Augmentation, Decomposition, and Procrustes (OADP) transformation. Estimated shrinkage coefficients (comparing red and blue points) for the first 20 PCs are 1.01 (PC1), 1.02, 1.06, 1.08, 1.36 (PC5), 1.82, 2.33, 2.36, 2.78, 2.84 (PC10), 2.99, 3.51, 4.38, 4.67, 4.99, 5.31, 5.74, 6.55, 6.71 and 6.75 (PC20). Note that only 20,000 random projected individuals are represented in this plot.

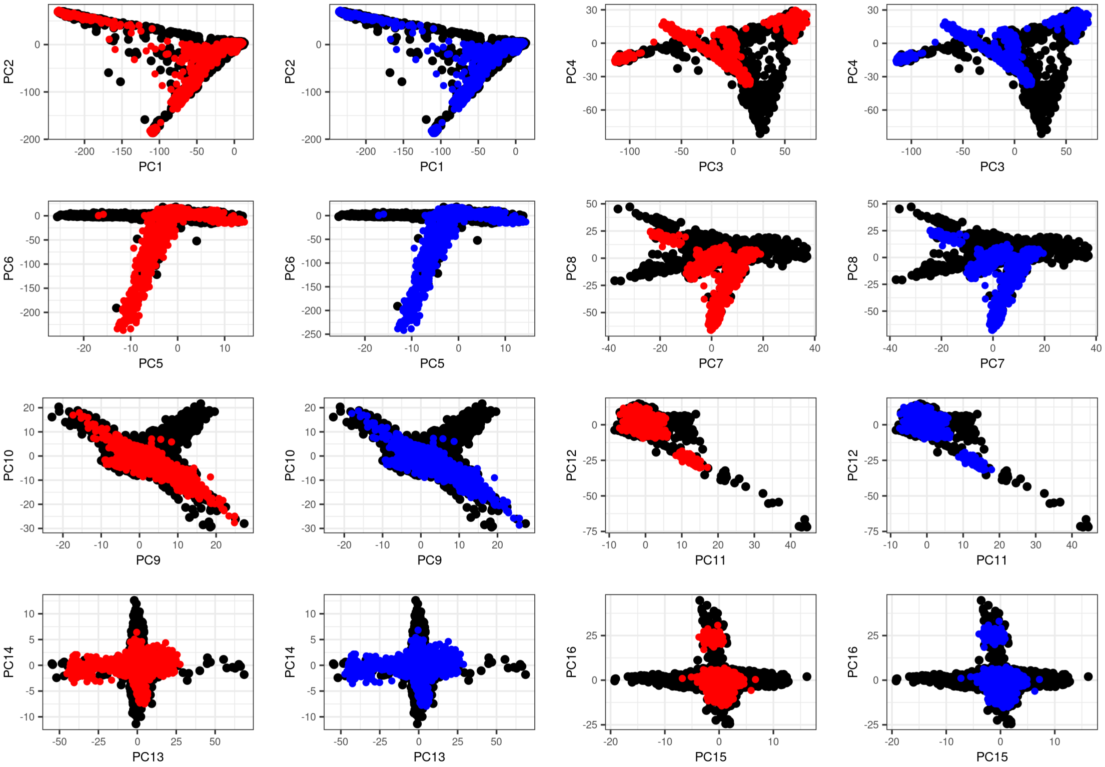


Figure S7: Principal Component (PC) scores 1 to 16 from the UK Biobank and projected individuals of the 1000 Genomes (1000G) project. Black points are the UK Biobank individuals used for computing PCA. Red points are the individuals from 1000G, projected by simply multiplying their genotypes by the corresponding PC loadings. Blue points are the individuals from 1000G, projected using the Online Augmentation, Decomposition, and Procrustes (OADP) transformation. Estimated shrinkage coefficients (comparing red and blue points) for the first 20 PCs are 1.00 (PC1), 1.00, 1.00, 1.01, 1.01 (PC5), 1.02, 1.03, 1.03, 1.04, 1.04 (PC10), 1.04, 1.05, 1.05, 1.06, 1.07, 1.07, 1.08, 1.08, 1.08 and 1.08 (PC20).

# PCA of the UK Biobank

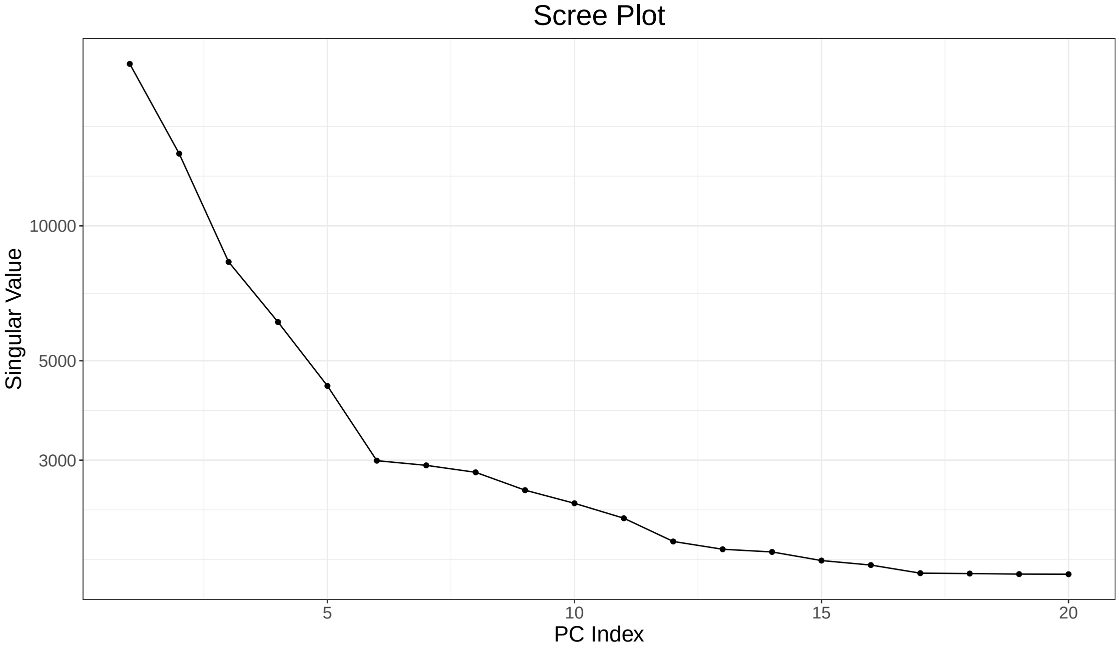


Figure S8: Scree plot: plot of singular values computed on the UK Biobank using `bed_autoSVD`.

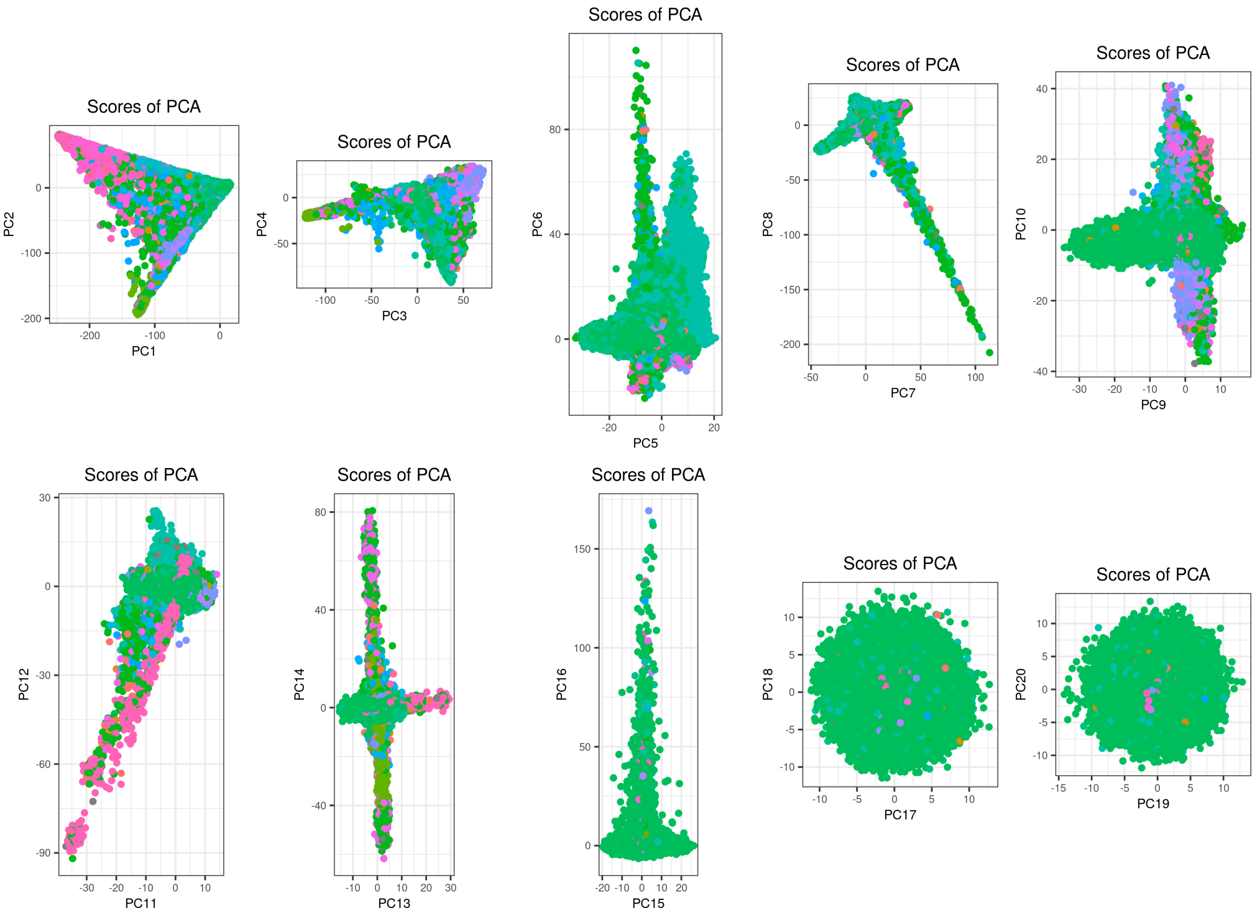


Figure S9: Principal Component (PC) scores 1 to 20 computed on the UK Biobank using `bed_autoSVD`. Different colors represent different self-reported ancestries.

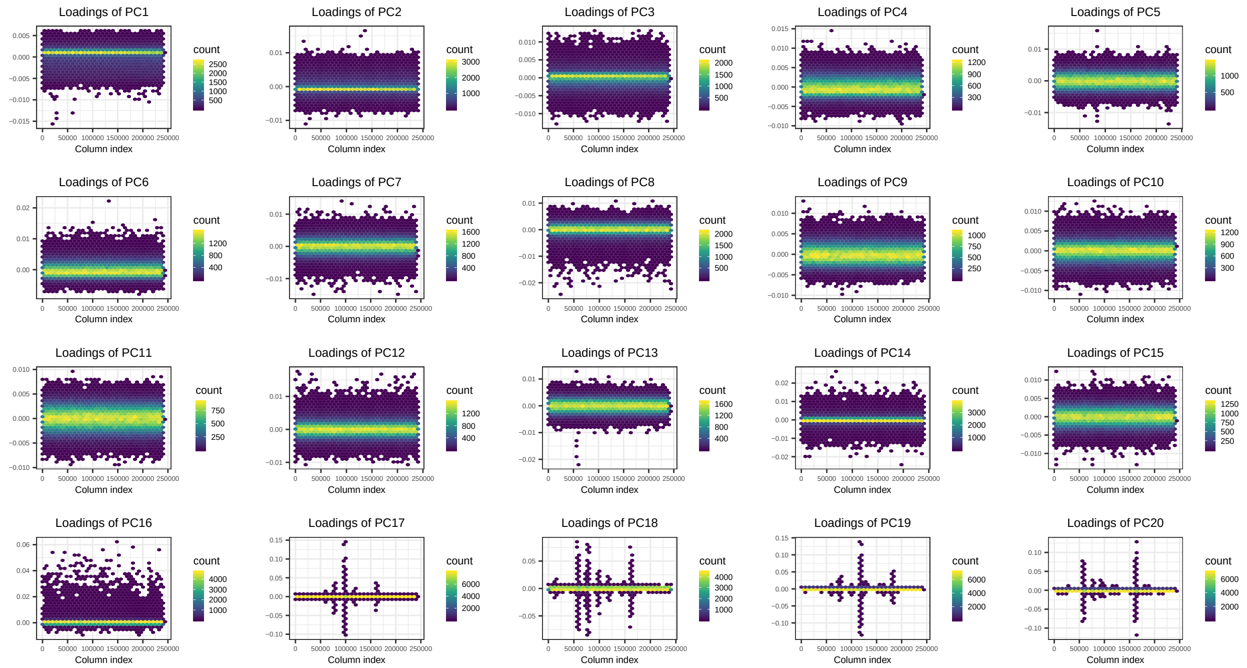


Figure S10: Principal Component (PC) loadings 1 to 20 computed on the UK Biobank using `bed_autoSVD`. Column indices of variants in the data, ordered by chromosome and physical position, are represented on the x-axis, and the value of loadings are represented on the y-axis. Points are hex-binned.

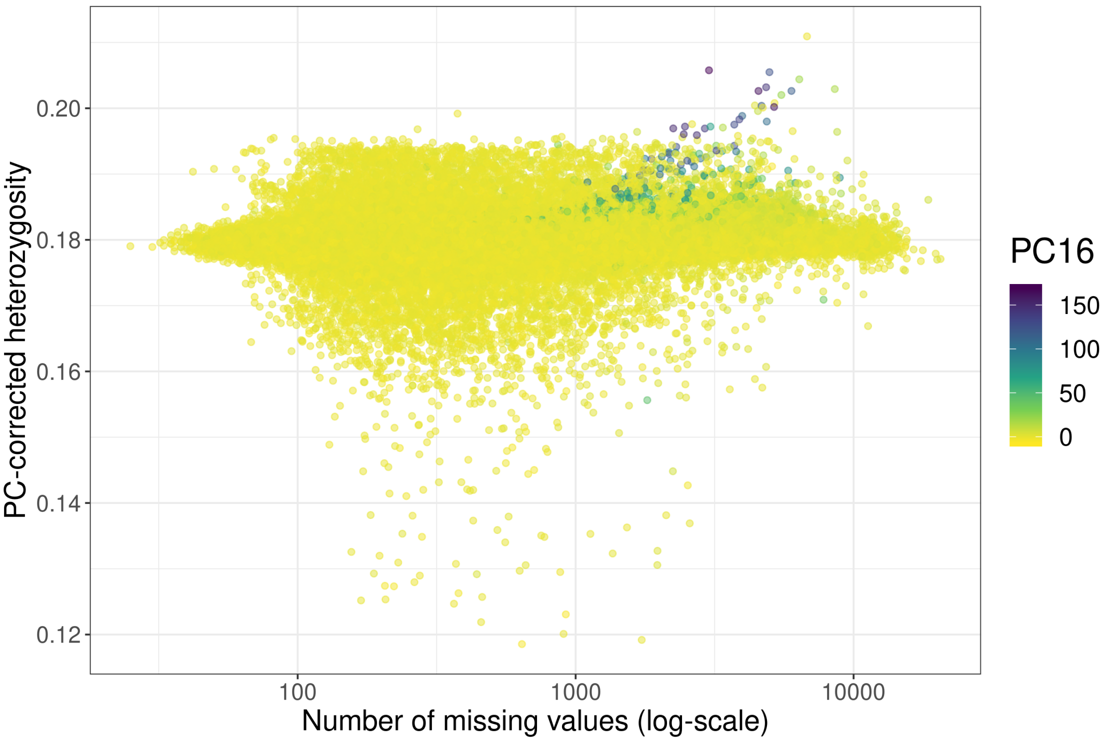


Figure S11: PC-corrected heterozygosity and number of missing values for individuals of the UK Biobank, colored by their value for PC16.

Table S2: Correlation between first 20 PC scores of the UK Biobank, both computed using the genotyping chip and mean imputation, but wtih either no quality control on individuals based on high heterozygosity (top), or after removing some individuals based on high heterozygosity (left). PC16 computed without quality control on high heterozygosity completely disappears when performing this quality control.

| | 1 | 2 | 3 | 4 | 5 | 6 | 7 | 8 | 9 | 10 | 11 | 12 | 13 | 14 | 15 | 16 | 17 | 18 | 19 | 20 |
|---|---|---|---|---|---|---|---|---|---|---|---|---|---|---|---|---|---|---|---|---|
| 1 | 100.0 | -0.2 | 0.1 | 0.0 | 0.0 | 0.0 | 0.0 | 0.0 | -0.0 | -0.0 | 0.0 | 0.0 | -0.0 | -0.0 | -0.0 | -0.3 | -0.0 | 0.0 | 0.0 | 0.0 |
| 2 | 0.1 | 100.0 | 0.2 | 0.0 | 0.0 | 0.0 | 0.0 | 0.0 | 0.0 | -0.1 | 0.0 | -0.0 | 0.0 | -0.0 | -0.0 | 0.0 | -0.0 | -0.0 | 0.0 | 0.0 |
| 3 | -0.0 | -0.1 | 100.0 | -0.0 | 0.0 | -0.0 | -0.0 | -0.0 | -0.0 | 0.1 | -0.0 | 0.0 | 0.0 | -0.0 | -0.0 | -0.3 | 0.0 | 0.0 | -0.0 | -0.0 |
| 4 | -0.0 | -0.0 | 0.0 | 100.0 | -0.0 | 0.0 | 0.0 | 0.0 | -0.0 | -0.0 | 0.0 | 0.0 | -0.0 | -0.0 | -0.0 | 0.1 | 0.0 | 0.0 | 0.0 | 0.0 |
| 5 | 0.0 | -0.0 | -0.0 | 0.0 | 100.0 | 0.0 | -0.0 | 0.0 | 0.0 | -0.0 | -0.0 | 0.0 | 0.0 | 0.0 | -0.0 | 0.1 | -0.0 | -0.0 | -0.0 | 0.0 |
| 6 | -0.0 | -0.0 | 0.0 | -0.0 | -0.0 | 100.0 | -0.3 | 0.4 | -0.0 | 0.0 | -0.0 | 0.0 | -0.0 | -0.0 | 0.0 | -0.1 | 0.0 | -0.0 | -0.0 | 0.0 |
| 7 | -0.0 | -0.0 | 0.0 | -0.0 | 0.0 | 0.3 | 100.0 | -0.2 | -0.0 | 0.2 | -0.1 | -0.1 | 0.0 | 0.0 | 0.0 | 0.3 | -0.0 | -0.0 | 0.0 | -0.0 |
| 8 | -0.0 | -0.0 | 0.0 | -0.0 | -0.0 | -0.4 | 0.2 | 100.0 | 0.0 | 0.2 | -0.1 | -0.0 | 0.0 | 0.0 | 0.0 | 0.2 | 0.0 | 0.0 | -0.0 | 0.0 |
| 9 | 0.0 | -0.0 | 0.0 | 0.0 | -0.0 | 0.0 | 0.0 | -0.0 | 100.0 | 0.2 | -0.0 | 0.0 | -0.0 | -0.0 | 0.0 | -0.5 | -0.0 | 0.0 | -0.0 | 0.0 |
| 10 | 0.0 | 0.0 | -0.0 | 0.0 | 0.0 | -0.0 | -0.1 | -0.1 | -0.2 | 100.0 | 0.4 | -0.1 | 0.1 | 0.0 | 0.0 | 0.8 | -0.0 | -0.0 | 0.0 | 0.0 |
| 11 | -0.0 | -0.0 | 0.0 | -0.0 | 0.0 | 0.0 | 0.0 | 0.0 | 0.0 | -0.3 | 100.0 | 0.2 | -0.1 | -0.0 | -0.0 | -0.2 | 0.0 | 0.0 | -0.0 | 0.0 |
| 12 | -0.0 | 0.0 | -0.0 | -0.0 | -0.0 | -0.0 | 0.0 | 0.0 | -0.0 | 0.1 | -0.1 | 100.0 | -0.3 | 0.1 | 0.0 | 1.7 | -0.0 | -0.1 | -0.0 | -0.0 |
| 13 | 0.0 | -0.0 | -0.0 | 0.0 | -0.0 | 0.0 | -0.0 | -0.0 | 0.0 | -0.0 | 0.0 | 0.3 | 100.0 | -0.4 | -0.1 | -2.8 | 0.0 | 0.0 | 0.1 | 0.0 |
| 14 | 0.0 | 0.0 | 0.0 | 0.0 | -0.0 | 0.0 | -0.0 | -0.0 | -0.0 | -0.0 | 0.0 | -0.1 | 0.4 | 100.0 | -0.0 | -1.8 | 0.0 | 0.0 | 0.0 | -0.0 |
| 15 | 0.0 | 0.0 | -0.0 | 0.0 | 0.0 | -0.0 | 0.0 | -0.0 | -0.0 | -0.0 | 0.0 | -0.0 | 0.0 | -0.0 | 100.0 | -2.1 | 0.1 | -0.0 | 0.0 | 0.0 |
| 16 | 0.0 | 0.0 | -0.0 | -0.0 | 0.0 | -0.0 | 0.0 | -0.0 | 0.0 | 0.0 | -0.0 | 0.0 | 0.0 | 0.0 | -0.0 | 1.3 | 100.0 | 0.6 | -0.1 | -0.3 |
| 17 | -0.0 | -0.0 | -0.0 | 0.0 | -0.0 | -0.0 | -0.0 | 0.0 | -0.0 | -0.0 | 0.0 | 0.0 | -0.0 | -0.0 | -0.1 | -5.0 | 0.7 | -99.9 | -0.8 | -0.2 |
| 18 | 0.0 | 0.0 | -0.0 | 0.0 | -0.0 | -0.0 | 0.0 | 0.0 | -0.0 | 0.0 | -0.0 | 0.0 | -0.0 | -0.0 | -0.0 | -2.9 | 0.0 | 0.9 | -99.5 | 8.7 |
| 19 | 0.0 | 0.0 | -0.0 | 0.0 | 0.0 | 0.0 | -0.0 | 0.0 | 0.0 | 0.0 | 0.0 | -0.0 | 0.0 | -0.0 | 0.0 | -0.3 | -0.2 | 0.3 | -8.6 | -99.6 |
| 20 | 0.0 | 0.0 | -0.0 | 0.0 | -0.0 | -0.0 | -0.0 | 0.0 | 0.0 | 0.0 | 0.0 | -0.0 | 0.0 | 0.0 | 0.1 | 7.3 | -0.8 | -0.3 | -4.4 | -0.3 |

Table S3: Correlation between first 20 PC scores of the UK Biobank, either computed using the genotyping chip and mean imputation (top), or computed from the dosages (based on imputation from an external reference panel) of the same variants (left). PCs are globally the same.

| | 1 | 2 | 3 | 4 | 5 | 6 | 7 | 8 | 9 | 10 | 11 | 12 | 13 | 14 | 15 | 16 | 17 | 18 | 19 | 20 |
|---|---|---|---|---|---|---|---|---|---|---|---|---|---|---|---|---|---|---|---|---|
| 1 | -100.0 | -0.1 | -0.1 | 0.0 | -0.0 | 0.0 | -0.0 | -0.0 | -0.0 | 0.0 | -0.0 | -0.0 | -0.0 | -0.0 | -0.0 | -0.0 | 0.0 | 0.0 | 0.0 | -0.0 |
| 2 | 0.1 | -100.0 | -0.2 | -0.0 | -0.0 | -0.0 | 0.0 | -0.0 | 0.0 | -0.0 | 0.0 | 0.0 | 0.0 | -0.0 | 0.0 | 0.0 | -0.0 | -0.0 | -0.0 | 0.0 |
| 3 | -0.1 | -0.2 | 100.0 | -0.2 | 0.0 | 0.0 | 0.1 | 0.0 | 0.0 | 0.0 | -0.0 | 0.0 | 0.0 | -0.0 | -0.0 | 0.0 | 0.0 | -0.0 | 0.0 | -0.0 |
| 4 | 0.0 | -0.0 | 0.2 | 100.0 | 0.1 | 0.0 | -0.1 | -0.0 | 0.0 | 0.1 | -0.0 | 0.0 | 0.0 | 0.0 | 0.0 | 0.0 | -0.0 | 0.0 | -0.0 | 0.0 |
| 5 | 0.0 | 0.0 | 0.0 | 0.1 | -100.0 | 0.0 | -0.0 | -0.0 | 0.0 | 0.0 | -0.0 | -0.0 | 0.0 | 0.0 | 0.0 | -0.0 | 0.0 | 0.0 | -0.0 | -0.0 |
| 6 | -0.0 | -0.0 | -0.0 | -0.0 | 0.0 | 99.9 | -2.7 | 2.0 | -0.0 | -0.0 | -0.0 | 0.0 | -0.0 | 0.0 | 0.0 | -0.0 | 0.0 | 0.0 | -0.0 | -0.0 |
| 7 | -0.0 | -0.0 | -0.1 | 0.1 | -0.0 | 2.6 | 99.9 | 3.3 | -0.1 | -0.1 | 0.0 | -0.0 | 0.0 | 0.0 | -0.0 | -0.0 | 0.0 | -0.0 | 0.0 | -0.0 |
| 8 | 0.0 | 0.0 | -0.0 | 0.0 | -0.0 | -2.1 | -3.2 | 99.9 | 0.2 | -0.2 | 0.0 | -0.0 | -0.0 | -0.0 | -0.0 | -0.0 | -0.0 | -0.0 | 0.0 | -0.0 |
| 9 | -0.0 | -0.0 | -0.0 | -0.0 | -0.0 | 0.0 | 0.1 | -0.2 | 100.0 | -0.0 | -0.0 | -0.0 | -0.0 | -0.0 | 0.0 | -0.0 | 0.0 | 0.0 | 0.0 | 0.0 |
| 10 | -0.0 | 0.0 | 0.1 | 0.0 | -0.0 | -0.0 | -0.1 | -0.1 | -0.0 | -99.9 | -0.3 | -0.1 | 0.0 | -0.0 | 0.0 | -0.0 | -0.0 | -0.0 | -0.0 | -0.0 |
| 11 | 0.0 | -0.0 | -0.0 | -0.0 | 0.0 | -0.0 | 0.0 | 0.0 | -0.0 | 0.4 | -99.9 | 0.2 | 0.1 | 0.0 | 0.0 | -0.0 | -0.0 | -0.0 | 0.0 | -0.0 |
| 12 | 0.0 | -0.0 | -0.0 | 0.0 | -0.0 | 0.0 | -0.0 | -0.0 | -0.0 | 0.1 | -0.2 | -99.9 | 0.7 | -0.0 | 0.1 | 0.0 | 0.0 | 0.0 | 0.0 | 0.0 |
| 13 | -0.0 | 0.0 | -0.0 | -0.0 | -0.0 | 0.0 | -0.0 | 0.0 | 0.0 | 0.0 | 0.1 | 0.7 | 99.9 | 2.1 | 0.0 | -0.0 | 0.0 | -0.1 | -0.0 | 0.0 |
| 14 | -0.0 | -0.0 | 0.0 | 0.0 | 0.0 | -0.0 | -0.0 | 0.0 | 0.0 | -0.0 | 0.0 | -0.0 | -2.1 | 99.9 | -0.1 | -0.0 | 0.0 | -0.0 | 0.0 | 0.0 |
| 15 | -0.0 | -0.0 | -0.0 | 0.0 | 0.0 | -0.0 | -0.0 | 0.0 | -0.0 | 0.0 | 0.0 | 0.1 | -0.0 | 0.1 | 99.9 | 0.1 | 0.0 | -0.1 | 0.1 | 0.1 |
| 16 | -0.0 | 0.0 | -0.0 | 0.0 | 0.0 | -0.0 | -0.0 | -0.0 | -0.0 | -0.0 | -0.0 | -0.0 | -0.0 | -0.0 | 0.0 | -99.7 | 6.4 | 0.8 | 0.9 | -1.3 |
| 17 | -0.0 | 0.0 | -0.0 | 0.0 | 0.0 | -0.0 | 0.0 | 0.0 | 0.0 | 0.0 | -0.0 | 0.0 | -0.0 | -0.0 | -0.0 | 6.4 | 99.6 | -1.0 | 2.4 | 0.5 |
| 18 | 0.0 | -0.0 | -0.0 | 0.0 | -0.0 | -0.0 | 0.0 | -0.0 | 0.0 | 0.0 | 0.0 | -0.0 | -0.1 | -0.0 | -0.1 | -0.9 | -0.9 | -99.6 | -5.0 | 4.0 |
| 19 | 0.0 | -0.0 | -0.0 | 0.0 | 0.0 | -0.0 | 0.0 | 0.0 | 0.0 | 0.0 | 0.0 | -0.0 | -0.0 | 0.0 | 0.1 | -0.6 | 2.5 | 4.9 | -99.5 | -6.2 |
| 20 | -0.0 | 0.0 | 0.0 | 0.0 | -0.0 | -0.0 | 0.0 | 0.0 | -0.0 | -0.0 | -0.0 | 0.0 | -0.0 | -0.0 | -0.0 | -1.4 | -0.2 | 4.3 | -6.0 | 99.5 |

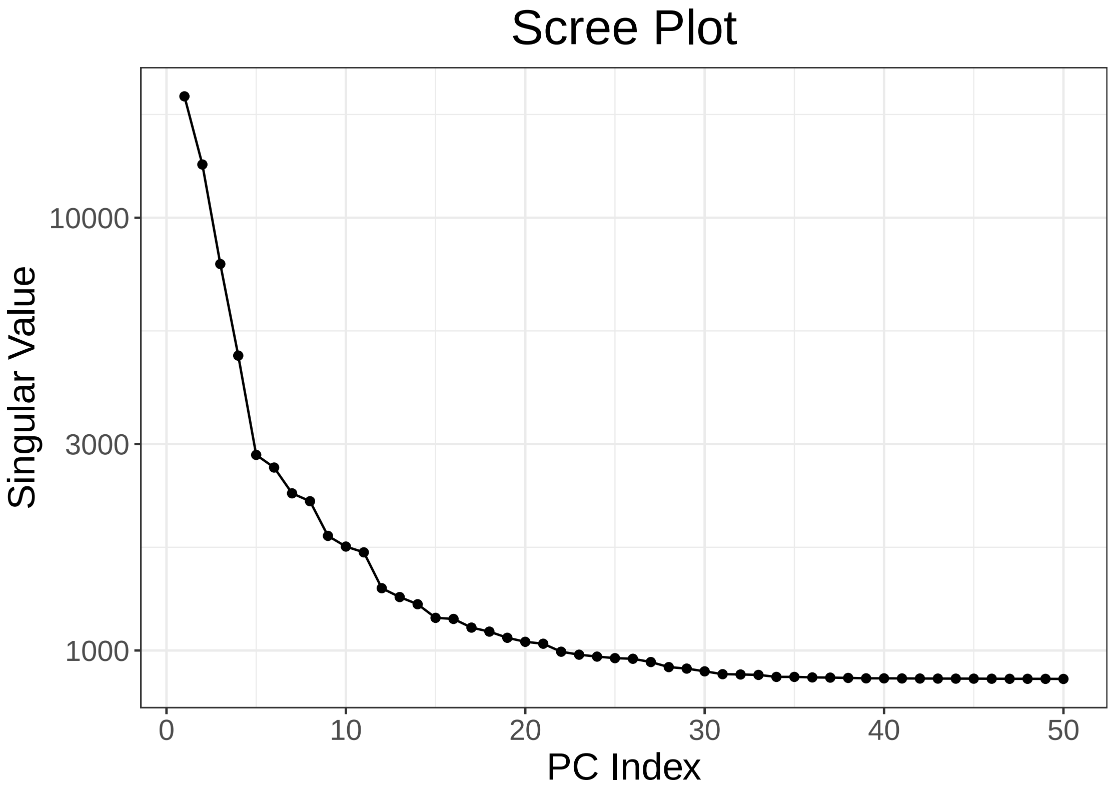


Figure S12: Scree plot: plot of singular values computed on the UK Biobank using 48,942 individuals of diverse ancestries. These individuals are the ones resulting from removing all related individuals and randomly subsampling the British and Irish individuals.

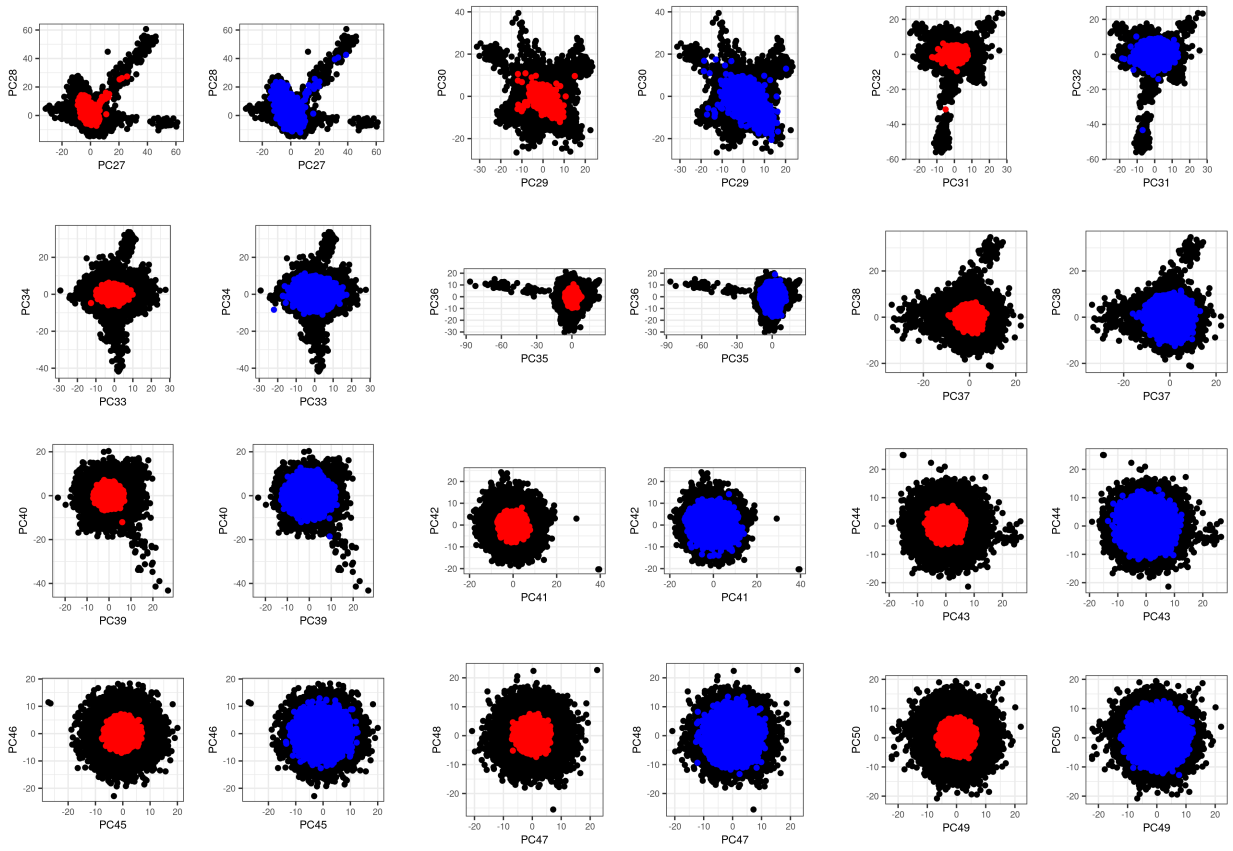

Figure S13: Principal Component (PC) scores 27 to 50 of the UK Biobank. Black points are the 48,942 individuals of diverse ancestries used for computing PCA. These individuals are the ones resulting from removing all related individuals and randomly subsampling the British and Irish individuals. Red points are the remaining UKBB individuals, projected by simply multiplying their genotypes by the corresponding PC loadings. Blue points are the remaining UKBB individuals, projected using the Online Augmentation, Decomposition, and Procrustes (OADP) transformation. Note that only 20,000 random projected individuals are represented in this plot.
